# Ternary Mixtures of Hard Spheres and Their Multiple Separated Phases

**DOI:** 10.3390/molecules28237817

**Published:** 2023-11-28

**Authors:** Luka Sturtewagen, Erik van der Linden

**Affiliations:** Laboratory of Physics and Physical Chemistry of Foods, Wageningen University, Bornse Weilanden 9, 6708 WG Wageningen, The Netherlands

**Keywords:** polydispersity, hard spheres, phase behavior, virial coefficient

## Abstract

We study the liquid phase behavior of ternary mixtures of monodisperse hard spheres in solution. The interactions are modeled in terms of the second virial coefficient and can be additive hard sphere (HS) or non-additive hard sphere (NAHS) interactions. We give the set of equations that defines the phase diagram for mixtures of three components. We calculate the theoretical liquid–liquid phase separation boundary for two-phase separation (the binodal) and, if applicable, the three-phase boundary, as well as the plait points and the spinodal. The sizes of the three components are fixed. The first component (A) is the smallest one, the second component (B) is four times the size of the smallest component, and the third (C) component is three times the size of the smallest one. The interaction between the first two components is fixed, and this AB sub-mixture shows phase separation. The interactions of component C with the other two components are varied. Component C can be compatible or incompatible with components A and B. Depending on the compatibility of the components, the phase diagram is altered. The addition of the third component has an influence on the phase boundary, plait points, stability region, fractionation, and volume ratio between the different phases. When all sub-mixtures (AB, AC, and BC) show phase separation, a three-phase system becomes possible when the incompatibility among all components is high enough. The position and size of the three-phase region is dependent on the interactions between the different sub-mixtures. We study the fractionation off all components depending on specific parent concentrations.

## 1. Introduction

A lot of research, both theoretical and experimental, has been focused on the phase behavior of two components in solution. However, most products consist of more components. The investigation of multicomponent phase behavior has a considerable number of challenges. From an experimental point of view, the number of samples to prepare to obtain an insight into the phase behavior at different concentrations in the phase diagram increases significantly with the number of considered components. From a theoretical point of view, the number of pairwise interactions for a mixture with n components taken into account increases according to n(n + 1)/2. The number of interactions increases even more when higher-order interactions are taken into account. Depending on the interactions between all the components, the theoretical number of possible phases that can be formed will increase as well, as follows from the Gibbs phase rule. The Gibbs phase rule gives the relationship between the number of possible phases and the degrees of freedom (dof) for a system: dof = n + 2 − f, in which n is the number of components and f is the maximum number of phases in equilibrium. The degrees of freedom for the system are, for example, the mole fraction of the components, the temperature, or the pressure. When looking at multicomponent systems, one may distinguish chemically different components and chemically equivalent components that only differ in size. The latter are often met in experimental conditions since many components show polydispersity in their size (e.g., mixtures containing polymers such as poly-ethylene glycol, dextran, gelatin, etc.) [1,2,3,4]. In this work, we will focus on mixtures containing three distinct types of hard spheres in solution with non-additive pair-wise interactions. They can be seen as a model for a system containing different components (e.g., polymers or colloids in solution).

Depletion interactions in systems with more than two components become more complicated than for a two-component system. This has been shown by Ji and Walz [5], who investigated the depletion interaction in a highly asymmetric ternary system. They found out that the energy profile between two particles is significantly altered due to the addition of bi-disperse depletants: the pair-potential has an additional attractive energy well and an extended repulsive barrier that are of significant magnitude, altering the stability of the dispersion of the larger particles. Park and Conrad [6] also studied the depletion interaction in a system of colloids and bi-disperse polymers. They report that the bi-disperse polymers could not be treated as homogeneous depletants. Both sizes of the polymers contributed to the effective interactions.

Some experimental work on mixtures with more than two components that demix into multiple-phase systems has focused on methods to aid in the partitioning of proteins or cell contents. Each of the phases in these systems has a different density and relatively low surface tension, aiding in the separation. An example of such a system is the system containing the high-molecular colloids dextran, ficoll, and poly-ethylene-glycol [7,8]. Similar results were obtained by Ruan et al. [9] for the system of the colloids dextran and polyethylene-glycol with the surfactant Triton X-100. Mixtures with an even higher number of components (both colloids and surfactants) and a corresponding higher number of phases in equilibrium were studied by others [10]. They report systems of up to six phases, each enriched in one of the polymers and depleted in the others.

A more in-depth experimental study on the phase behavior and phase diagram of a mixture of three components and the three-phase dynamics was performed by Beck-Candanedo et al. [11]. They studied the phase behavior of rods in the presence of two types of dextran. At high enough concentrations, rod-like particles such as cellulose nano-crystals form a two-phase system of an isotropic and an nematic phase. The addition of the different types of dextran leads to a widening of the two-phase coexistence region or the formation of an additional isotropic phase, resulting in a three-phase system.

The work on three-component systems paves the way for analysis of systems containing multiple-component systems. This may be important, for example, for cellular systems. Cells contain a large number of components in solution, and it has been hypothesized that demixing and phase separation leads to organization within cells [12]. It should be noted that the phase behavior will be further affected by the fact that a cell is a confined system and crowded by flexible particles. The confinement by the cell volume itself and the presence of the flexible particles both affect the phase diagram.

Another important application area is the phase behavior of practical industrial systems, such as in cosmetics and food systems, which consist of various types of polymers, each of which is, in fact, polydisperse (i.e., in effect forming a multi-component mixture).

For multi-component systems, Sear and Cuesta [13] investigated the instability in multicomponent mixtures, such as those present in cells. Their theory is also based on the second virial approximation, where all components are attributed virial coefficients and cross-virial coefficients in a random fashion, together with inserting a predefined distribution of all virial coefficients. They approximated the second virial coefficients of the pairwise interactions of the components in the system using random matrix theory. Their work yields insight into the nature of phase transitions occurring in such multicomponent mixtures (for example, whether one has demixing into phases where each of the components are spread out over the various phases, or whether all components are condensed into a phase that is separated from a diluted phase also containing all components).

In order to identify interesting characteristic behavior of such multicomponent systems, one could also attempt the same second-order virial approach, instead of randomly attributing virial coefficients as in the random matrix theory approach. One can then use experimentally accessible virial coefficients between the components and solve the equations numerically. Our work is aimed at this approach, starting with three-component systems in this article, which is worked out for mixtures with more components in detail elsewhere [14]. It is noted that the occasionally qualitative nature of insights, as also emerges from random matrix theory, will emerge from using other second-order virial approaches as well, in particular when applying it to non-dilute concentrations. We thus mainly aim at exploring the effects of polydispersity and accordingly changing interactions, and refrain from finding exact numbers for critical points and from finding exact locations of the spinodal and co-existence curves.

The descriptions of characteristic behavior, such as those by Sear and Cuesta [13], are different from accurately predicting the location of plait points, binodals, and spinodals. Indeed, comparing our data for a two-component mixture [15] as obtained via a second virial approach with those obtained by Dijkstra [16], and Hopkins and Schmidt [17], the critical points differ from our results. For example, using a size ratio of 0.1 at a given non-additivity parameter 0.2, Hopkins and Schmidt find the critical point coordinates (0.004;0.31), with a summed critical point fraction of 0.31, whereas our value [15] amounts to a summed value of 0.18. Dijkstra finds a summed critical point fraction of around 0.4 suing Monte Carlo simulations. Despite the discrepancies, the value of a second virial approach can be seen in gauging the effects of important changes in a system at low concentrations. As such, we think that the current work still will be helpful in identifying the nature and importance of polydispersity in multicomponent mixtures.

Interestingly, work has been done on ternary additive hard sphere systems [18], though this work contains the radial distribution functions but does not reveal phase diagrams or critical lines.

In our current study, we aim to obtain a better understanding of how the addition of a third component influences the phase behavior of two components that show phase separation. We will study the position of the phase separation boundary, the spinodal, the plait points, and the fractionation. We model the interactions between the components using a virial approach up to second order. Then, we describe the equations for the stability boundary and the spinodal, the equations for the plait point line, and finally the equations defining the phase boundary. With our expressions, we have enough to calculate the full phase diagram for a variety of mixtures. We do this until we reach a summed concentration of 0.5. We add a third component C to a binary mixture AB that phase separates. First, the component C is compatible with both A and B. In the second scenario we study, C is compatible with A or B, and incompatible with the other one. In the third scenario, C is incompatible with both A and B. Subsequently, we investigate the concentration of each component in the phase-separated mixtures at specific parent concentrations. In the last section, we compare three-component systems with a different particle size and different interactions that show three-phase separation.

## 2. Theory

We show the equations used for the calculations of the ternary phase diagram of the different studied systems: the set of equations defining the stability boundary, the plait points, and phase boundaries of a mixture. All sets of equations are solved in MATLAB R2017b. For a more detailed derivation of the equations, we refer to [19] and references therein. The set of equations determining the phase diagram originate from expressions for the osmotic pressure and chemical potentials, which should be equal among the coexisting phases in equilibrium.

### 2.1. Osmotic Virial Coefficient

The osmotic pressure, Π, of a solute in a fluid, at a temperature *T*, can be written as a virial expansion, similar to the virial expansion of the universal gas law for real gases [20]:

*β*Π = *ρ* + *B*_2_(*T*, *µ_s_*)*ρ*^2^ + *B*_3_(*T*, *µ_s_*)*ρ*^3^ + … (1)

where *β* = $\frac{1}{kT}$*; k* is Boltzmann’s constant; *T* the temperature; *ρ* the number density of the solute component, (N/V), with N the number particles, in volume *V*; and *B*_2_ and *B*_3_ the second and third virial coefficients of the solute component. The second virial coefficient accounts for the increase in osmotic pressure due to particle pairwise interaction. The third virial coefficient accounts for the interaction between three particles in a variety of configurations. The equation can be expanded for higher densities with *B_i_*, the *i*th virial coefficient of the solute, which accounts for the interaction between *i* different solute particles.

In this work, we will limit the virial expansion to the second virial coefficient, which is given by [21]:

(2) $$B_{2}\left(T,\mu_{s}\right)=2_{\;}\pi \int_{0}^{\infty }r^{2}(1-exp[-\beta W(r,\mu_{s})])\;dr$$

in which *µ_s_* is the chemical potential of the solvent, *W_ij_* (*r*) is the interaction potential between the particles *i* and *j*, and *r* is the distance between the center of the particles. For additive hard sphere (HS) interaction, the interaction potential for two particles (of the same species or different species) is, under constant chemical potential of the solvent, simplified to:

(3) $$W_{ij}\left(r\right){\;}_{HS}=\left\{\begin{array}{c} 0,\;\;\\ \infty , \end{array}\begin{array}{c} r\\ r \end{array}\right.\begin{array}{c} >\\ \leq \end{array}\;\begin{array}{c} \sigma_{ij}\\ \sigma_{ij} \end{array}$$

where *σ_ij_* = (*σ_i_* + *σ_j_*)/2, the distance between the centers of the two particles, and *σ_i_* and *σ_j_* are their respective diameters. The assumption of the chemical potential of the solvent remaining constant holds when the solute particles approach one another under the condition of constant osmotic pressure of the solution and constant partial volume of the solvent.

For non-additive hard spheres (NAHS), the distance of the closest approach of the centers of the two particles of different species can be closer or further than the distance between their centers [22]. The closest distance then becomes: *σ_ij_* = ((*σ_i_* + *σ_j_*)/2)(1 + ∆), in which ∆ (*≥−*1) accounts for the non-additivity of the interaction between the particles. When ∆ *>* 0, the distance of closest approach of both spheres increases, and when ∆ *<* 0, the distance of closest approach decreases compared to that due to HS interaction only. For additive hard sphere interaction, ∆ = 0. We note that for hard spheres and non-additive hard spheres, the virial coefficient does not depend on temperature, nor on the density of the spheres.

**Figure 1:**
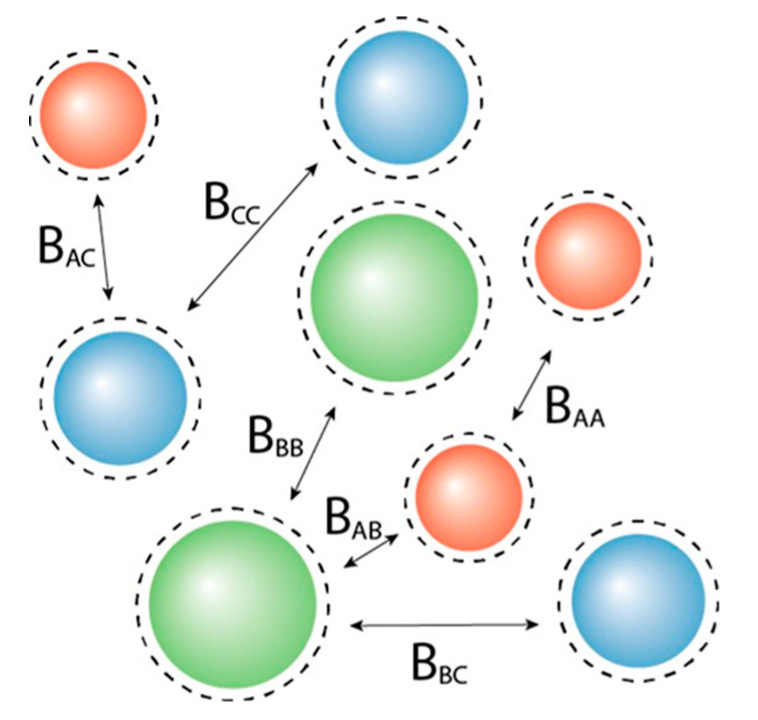
Graphical representation of a simple ternary mixture *ABC*. Second virial coefficients are indicated. The dotted lines around the spheres illustrate the effective radius of the particle, i.e., the actual radius increased by its non-additivity parameter D. Spheres that have the same color imply that these are equal to one another.

In a mixture with *n* distinguishable components in a solution, there are two main types of two-particle interactions that can occur: between particles of the same species and particles of different species. For the system we are studying, *n* = 3 (Figure 1). We refer in the text and in sub-indices to the three different components as A, B, and C. For the sake of brevity, in mathematical summations we use summation from 1 to 3 and, accordingly, indices i = 1, 2, or 3, with A being denoted by 1, B by 2, and C by 3. For clarity, we distinguish summations over the number of components, indicated by the summation from 1 to n, and for the number of phases, a summation from 1 to f, where f is the number of phases, which we consider in this article to be the phases I, II, or III.

For the second virial coefficient given by Equation (2), using the interaction potential defined in Equation (3), we find:

(4) $$B_{xx}=\frac{2\pi }{3}\left(\sigma_{x}\right){\;}^{3}$$

(5) $$B_{xy}=\frac{2\pi }{3}{\left(\left(\frac{\sigma_{x}+\sigma_{y}}{2}\right)\left(1+∆\right)\right)}^{3}$$

where *B_xx_* denotes the second virial coefficient for particles of the same species (assumed to be HS), and *B_xy_* the second virial coefficient of particles of different species, which can be HS or NAHS.

The general equation for the osmotic pressure for a dilute mixture of three components, A, B and C, limited to the second virial coefficient, is given by [19]:

(6) $$\beta II=\rho_{A}+\rho_{B}+\rho_{C}+B_{AA}\rho_{A}^{2}+{2B}_{AB}\rho_{A}\rho_{B}+{2B}_{AC}\rho_{A}\rho_{C}+B_{BB}\rho_{B}^{2}+{2B}_{BC}\rho_{B}\rho_{C}+B_{CC}\rho_{C}^{2}=\rho_{A}+\rho_{B}+\rho_{C}+\sum_{i}^{n}\;\sum_{j}^{n}\;B_{ij}\rho_{i}\rho_{j}$$

### 2.2. Stability of a Mixture

The differential of the free energy of a mixture is given by [20]:

(7) $$\;dA=-SdT-pdV+\sum_{i}^{n}\mu_{i}dN_{i}$$

in which *µ_i_*, the chemical potential (the first partial derivative of the free energy with respect to number of particles (*N_i_*)) for component *i*, is given by:

(8) $$\mu_{i}=\mu_{i}^{0}+kT\ln\left(\rho_{i}\right)+2kT\left(\sum_{j=1}^{n}B_{ij}\rho_{j}\right)$$

For a mixture with three distinguishable components, the second partial derivatives can be represented by a 3 × 3 matrix of the first partial derivatives of the chemical potential of each component.

This results in the following stability matrix:

(9) $$M_{1}=\left[\begin{array}{c} \frac{{\partial \mu }_{A}}{{\partial N}_{A}}\frac{{\partial \mu }_{A}}{{\partial N}_{B}}\frac{{\partial \mu }_{A}}{{\partial N}_{B}}\\ \frac{{\partial \mu }_{B}}{{\partial N}_{A}}\frac{{\partial \mu }_{B}}{{\partial N}_{B}}\frac{{\partial \mu }_{B}}{{\partial N}_{C}}\\ \frac{{\partial \mu }_{C}}{{\partial N}_{A}}\frac{{\partial \mu }_{C}}{{\partial N}_{B}}\frac{{\partial \mu }_{C}}{{\partial N}_{C}} \end{array}\right]=\left[\begin{array}{c} \begin{array}{c} {2B}_{AA}+\frac{1}{\rho_{A}}{\;\;\;\;\;\;\;\;\;\;\;\;2B}_{AB}{\;\;\;\;\;\;\;\;\;\;\;\;\;\;\;\;\;\;\;\;\;\;\;\;\;\;\;\;\;\;\;\;2B}_{AC}\\ {2B}_{AB}\;\;\;\;\;\;\;\;\;\;\;\;\;\;\;\;\;\;\;\;\;{2B}_{BB}+\frac{1}{\rho_{B}}{\;\;\;\;\;\;\;\;\;\;\;\;\;\;\;\;\;\;\;\;\;\;\;2B}_{BC} \end{array}\\ {\;\;\;\;\;\;\;\;\;2B}_{AC}{\;\;\;\;\;\;\;\;\;\;\;\;\;\;\;\;\;\;\;\;\;2B}_{BC}{\;\;\;\;\;\;\;\;\;\;\;\;\;\;\;\;\;\;\;\;\;\;\;\;\;\;\;\;\;\;\;\;2B}_{CC}+\frac{1}{\rho_{C}} \end{array}\right]$$

The mixture is stable when all eigenvalues are positive [23]. When, on the other hand, one of the eigenvalues is not positive, the mixture becomes unstable. The limit of stability is reached when the matrix has one zero eigenvalue and is otherwise positive definite, and it is referred to as the spinodal [24].

### 2.3. Plait Points

In a binary mixture, the critical point is a stable point that lies on the stability limit (spinodal) [24] and where the phase boundary and spinodal coincide. In mixtures of more components, these critical points become plait points. Critical points and plait points are generally concentrations at which two or more phases are in equilibrium and become indistinguishable [25,26].

There are two criteria that can be used to find plait points. The first one is *det*(*M*_1_) = 0, which is the equation for the spinodal. The other criterion is based on a characteristic at the critical point. For a multicomponent system, using Legendre transforms, one finds *det*(*M*_2_) = 0 [27,28], with:

(10) $$M_{2}=\left[\begin{array}{c} \frac{{\partial \mu }_{A}}{{\partial N}_{A}}\frac{{\partial \mu }_{A}}{{\partial N}_{B}}\frac{{\partial \mu }_{A}}{{\partial N}_{C}}\\ \frac{{\partial \mu }_{B}}{{\partial N}_{A}}\frac{{\partial \mu }_{B}}{{\partial N}_{B}}\frac{{\partial \mu }_{B}}{{\partial N}_{C}}\\ \frac{{\partial M}_{1}}{{\partial N}_{A}}\frac{{\partial M}_{1}}{{\partial N}_{B}}\frac{{\partial M}_{1}}{{\partial N}_{C}} \end{array}\right]$$

Matrix *M*_2_ is matrix *M*_1_ with one of the rows replaced by the partial derivatives of the determinant of matrix *M*_1_. Note that it does not matter which row of the matrix is replaced.

We find the following:

(11) $$M_{2}=\left[\begin{array}{ccc} {2B}_{AA}+\frac{1}{\rho_{A}} & {2B}_{AB} & {2B}_{AC}\\ {2B}_{AB} & {2B}_{BB}+\frac{1}{\rho_{B}} & {2B}_{BC}\\ \rho_{1} & \rho_{2} & \rho_{3} \end{array}\right]$$

with

$$P_{1}=-\frac{\left(\begin{array}{c} {-4B}_{BC}^{2}\rho_{B}\rho_{C}+{2B}_{BB}\rho_{B}+{2B}_{CC}\rho_{C}\\ +{4B}_{BB}B_{CC}\rho_{B}\rho_{C}+1 \end{array}\right)}{\rho_{A}^{2}\rho_{B}\rho_{C}}$$

$$P_{2}=-\frac{\left(\begin{array}{c} {-4B}_{AC}^{2}\rho_{A}\rho_{C}+{2B}_{AA}\rho_{A}+{2B}_{CC}\rho_{C}\\ +{4B}_{AA}B_{CC\rho A\rho C}+1 \end{array}\right)}{\rho_{A}\rho_{B}^{2}\rho_{C}}$$

$$P_{3}=-\frac{\left(\begin{array}{c} {-4B}_{AB}^{2}\rho_{A}\rho_{B}+{2B}_{AA}\rho_{A}+{2B}_{BB\rho B}\\ +{4B}_{AA}B_{BB\rho A\rho B}+1 \end{array}\right)}{\rho_{A}\rho_{B}\rho_{C}^{2}}$$

In general, *P_i_* can be found using the following equation [19]:

(12) $$P_{i}=-\frac{1}{\rho_{i}^{2}}M_{1,\left(ii\right)}$$

where *M*_1,(*ii*)_ is the minor of matrix *M*_1_ at the *i*th-row and *i*th-column.

We note that for some of the mixtures, the set of equations (*det*(*M*_1_) = 0 and *det*(*M*_2_) = 0), especially when all three components are incompatible, has solutions that are not on a phase boundary. We consider this a mathematical solution to the set of equations, but not a physical solution for a plait point, since plait points are defined to be concentrations on the phase boundary where two or more phases become indistinguishable. Therefore, these solutions will be ignored when depicting the phase diagram.

### 2.4. Phase Boundary

When a mixture becomes unstable and demixes into two or more phases, the chemical potential of each component and the osmotic pressure is the same in all phases [20], yielding:

(13) $$\;\left\{\begin{array}{c} {\beta II}^{I}={\beta II}^{II}=\cdots \\ \beta \mu_{1}^{I}={\beta \mu }_{1}^{II}=\cdots \\ \beta \mu_{n}^{I}=\beta \mu_{n}^{II}=\cdots \end{array}\right.$$

where the phases are denoted by *I*, *II*, and so on. For a mixture containing three distinguishable components that demixes into two phases, this results in 4 equations and 2 × 3 unknowns. If the mixture demixes into three phases, this results in 2 × 3 + 2 equations and 3 × 3 unknowns. To solve the set of equations without having to fix the concentration of one component and the ratio between the other components for at least one of the phases, we need extra equations. For the extra set of equations, we build on the fact that no particles are lost and no new particles are created during phase separation, and the fact that we assume that the total volume does not change.

For a system that separates into three phases, denoted by I, II, and III, we thus have the condition that the total number of particles, summed over the three different phases, I, II, and III, is constant, for each component A, B, and C. This condition for A, B and C, together with Equation (13), results in the following set of equations [19]:

(14) $$\;\;\left\{\begin{array}{c} \beta {II}^{I}=\beta {II}^{II}=\cdots \\ \beta \mu_{B}^{I}=\beta \mu_{B}^{II}=\cdots \\ \beta \mu_{B}^{I}=\beta \mu_{B}^{II}=\cdots \\ \beta \mu_{C}^{I}=\beta \mu_{C}^{II}=\cdots \\ \rho_{1}=\alpha^{I}\rho_{A}^{I}+\alpha^{II}\rho_{A}^{II}+\left(1-\alpha^{I}-\alpha^{II}\right)\rho_{A}^{III}\\ \rho_{2}=\alpha^{I}\rho_{B}^{I}+\alpha^{II}\rho_{B}^{II}+\left(1-\alpha^{I}-\alpha^{II}\right)\rho_{B}^{III}\\ \rho_{3}=\alpha^{I}\rho_{C}^{I}+\alpha^{II}\rho_{C}^{II}+\left(1-\alpha^{I}-\alpha^{II}\right)\rho_{C}^{III} \end{array}\right.$$

where $\alpha^{I}=\frac{V^{I}}{\sum_{i}^{f}V^{i}}$, $\alpha^{I}=\frac{V^{I}}{\sum_{i}^{f}V^{i}}$, and $\alpha^{I}=\frac{V^{I}}{\sum_{i}^{f}V^{i}}$, in which $V^{i}$ represents the volume of phase I, II, or III.

With Equation (14), we have 2 × 3 + 1 unknowns and 2 × 3 + 1 equations for mixtures that separate into two phases. For mixtures that demix into three phases, we will have 3 × 3 + 2 unknowns and 3 × 3 + 2 equations. Therefore, the set of equations given by Equation (14) allows for calculating the concentration of each component in each of the phases for any given parent concentration.

## 3. Results and Discussion

We calculate the phase diagram for a variety of ternary mixture of hard spheres (types *A*, *B*, and *C*) with non-additive interactions. For all particles, the concentrations are expressed as a dimensionless parameter according to *η_i_* = *πρ*$\sigma_{i}^{3}/6$ where i refers to either *A*, *B*, or *C*. If we refer to *η*, we imply the sum over *η_i_* over each i that is relevant to the specific case.

**Figure 2:**
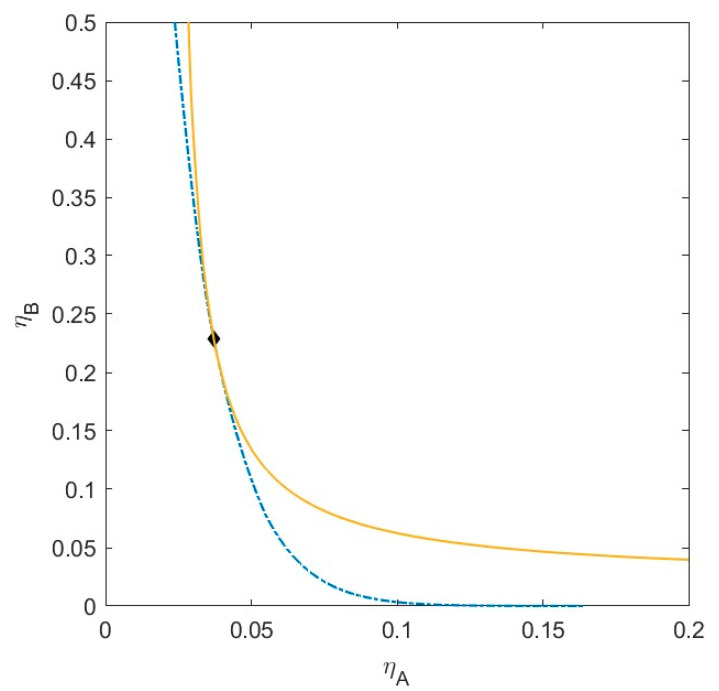
Phase diagram for monodisperse binary (components *A* and *B*) non-additive hard sphere mixture with size ratio *q* = *σ_A_*/*σ_B_* = 1/4 and ∆*_AB_* = 0.1, plotted as a function of the partial packing fractions, *η_A_* and *η_B_*. The spinodal (solid yellow line) and binodal (dashed blue line) meet each other at the critical point (black diamond).

**Figure 3:**
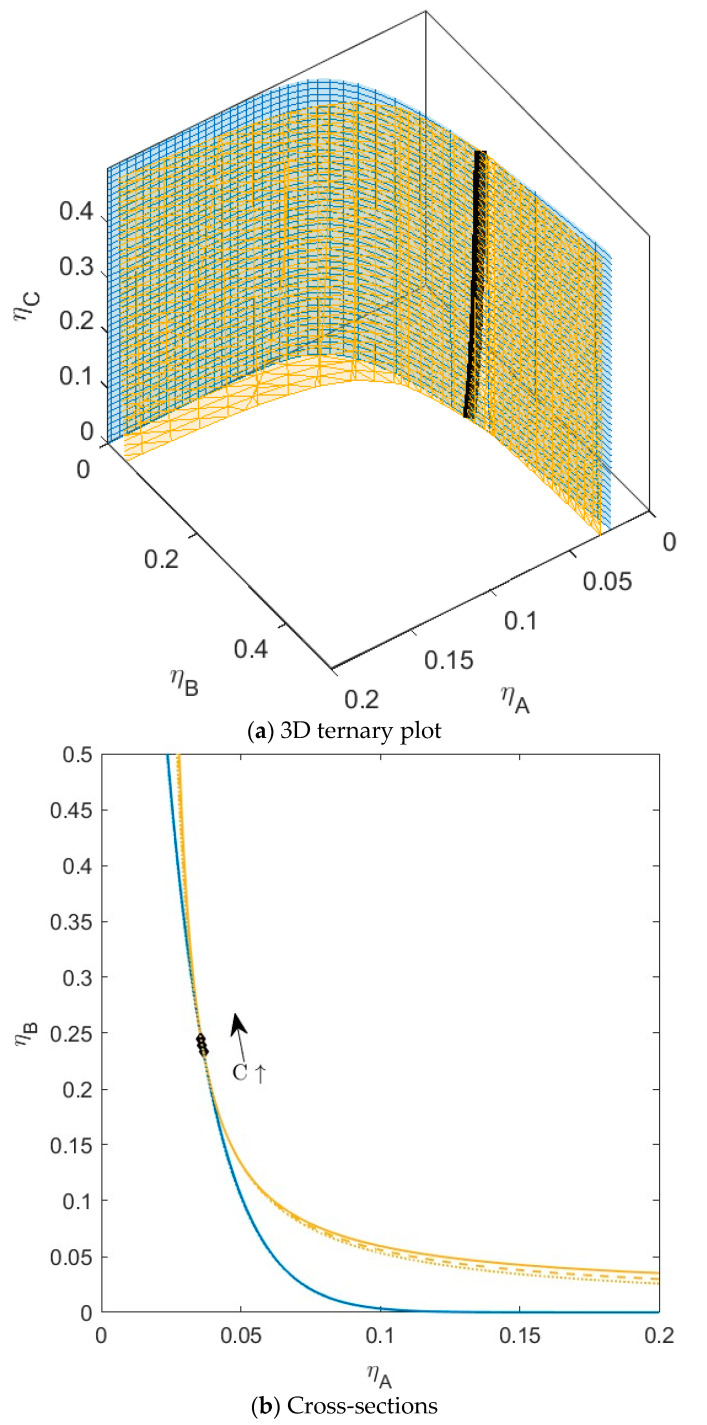
(**a**) Phase diagram for monodisperse ternary (components *A*, *B*, and *C*) non-additive hard sphere mixture with size ratio *q_AB_* = *σ_A_*/*σ_B_* = 1/4 and *q_AC_* = *σ_A_*/*σ_C_* = 1/3, with non-additivity parameters: ∆*_AB_* = 0.1, ∆*_AC_* = −0.1, and ∆*_BC_* = −0.1, plotted as a function of the partial packing fractions, *η_A_*, *η_B_*, and *η_C_*. The spinodal (yellow surface) and binodal (blue surface) meet each other at the plait point line (black line). (**b**) Phase diagram of component *A* and *B* at specific concentrations of component *C*; yellow lines are the spinodal, blue lines are the binodal, and the black diamonds are the critical points. Full line at concentration *η_C_* = 0.05, dashed line at concentration *η_C_* = 0.15, and dotted line at concentration *η_C_* = 0.30. With increasing concentrations of *C*, the critical point shifts to higher concentrations of *B.*

**Figure 4:**
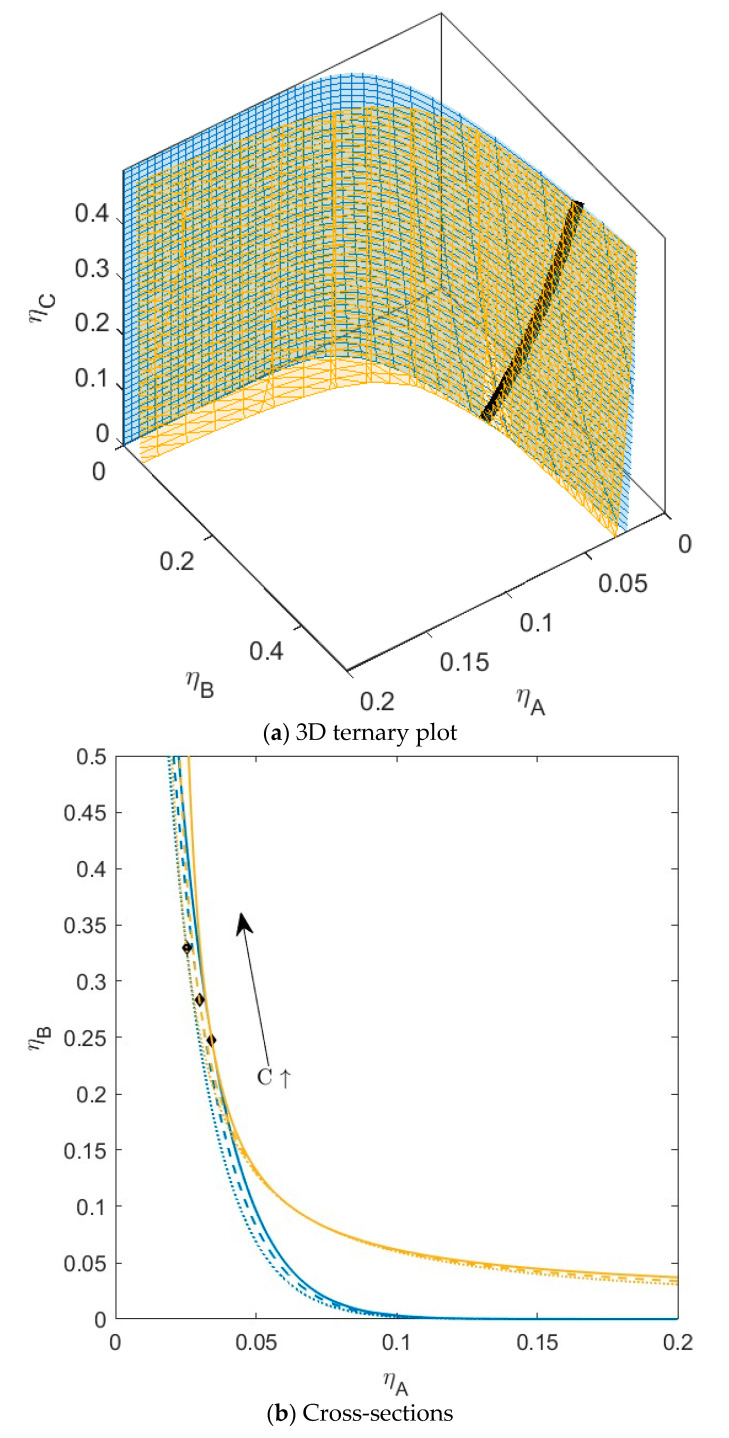
(**a**) Phase diagram for monodisperse ternary (components *A*, *B*, and *C*) non-additive hard sphere mixture with size ratio *q_AB_* = *σ_A_*/*σ_B_* = 1/4 and *q_AC_* = *σ_A_*/*σ_C_* = 1/3, with non-additivity parameters: ∆*_AB_* = 0.1, ∆*_AC_* = −0.1, and ∆*_BC_* = 0, plotted as a function of the partial packing fractions, *η_A_*, *η_B_*, and *η_C_*. The spinodal (yellow surface) and binodal (blue surface) meet each other at the plait point line (black line). (**b**) Phase diagram of component *A* and *B* at specific concentrations of component *C;* yellow lines are the spinodal, blue lines are the binodal, and the black diamonds are the critical points. Full line at concentration *η_C_* = 0.05, dashed line at concentration *η_C_* = 0.15, and dotted at concentration *η_C_* = 0.30. With increasing concentrations of *C*, the critical point shifts to higher concentrations of *B*.

**Figure 5:**
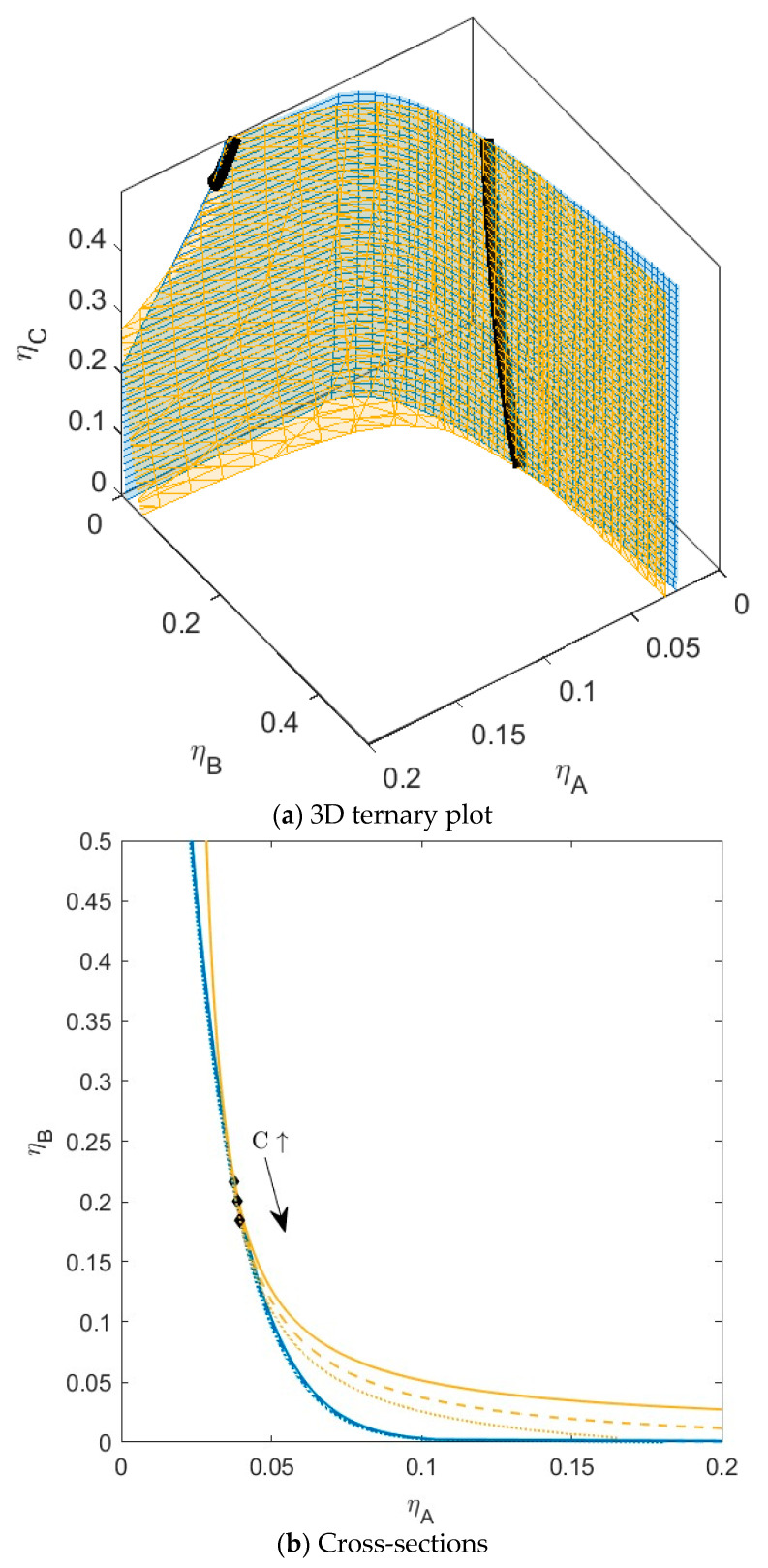
(**a**) Phase diagram for monodisperse ternary (components *A*, *B*, and *C*) non-additive hard sphere mixture with size atio *q_AB_* = *σ_A_*/*σ_B_* = 1/4 and *q_AC_* = *σ_A_*/*σ_C_* = 1/3, with non-additivity parameters: ∆*_AB_* = 0.1, ∆*_AC_* = 0, and ∆*_BC_* = −0.1, plotted as a function of the partial packing fractions, *η_A_*, *η_B_*, and *η_C_*. The spinodal (yellow surface) and binodal (blue surface) meet each other at the plait point line (black line). (**b**) Phase diagram of component *A* and *B* at specific concentrations of component *C*, yellow lines are the spinodal, blue lines are the binodal, and the black diamonds are the critical points. Full line at concentration *η_C_* = 0.05, dashed line at concentration *η_C_* = 0.15, and dotted line at concentration *η_C_* = 0.30. With increasing concentrations of *C*, the critical point shifts to lower concentrations of *B.*

**Figure 6:**
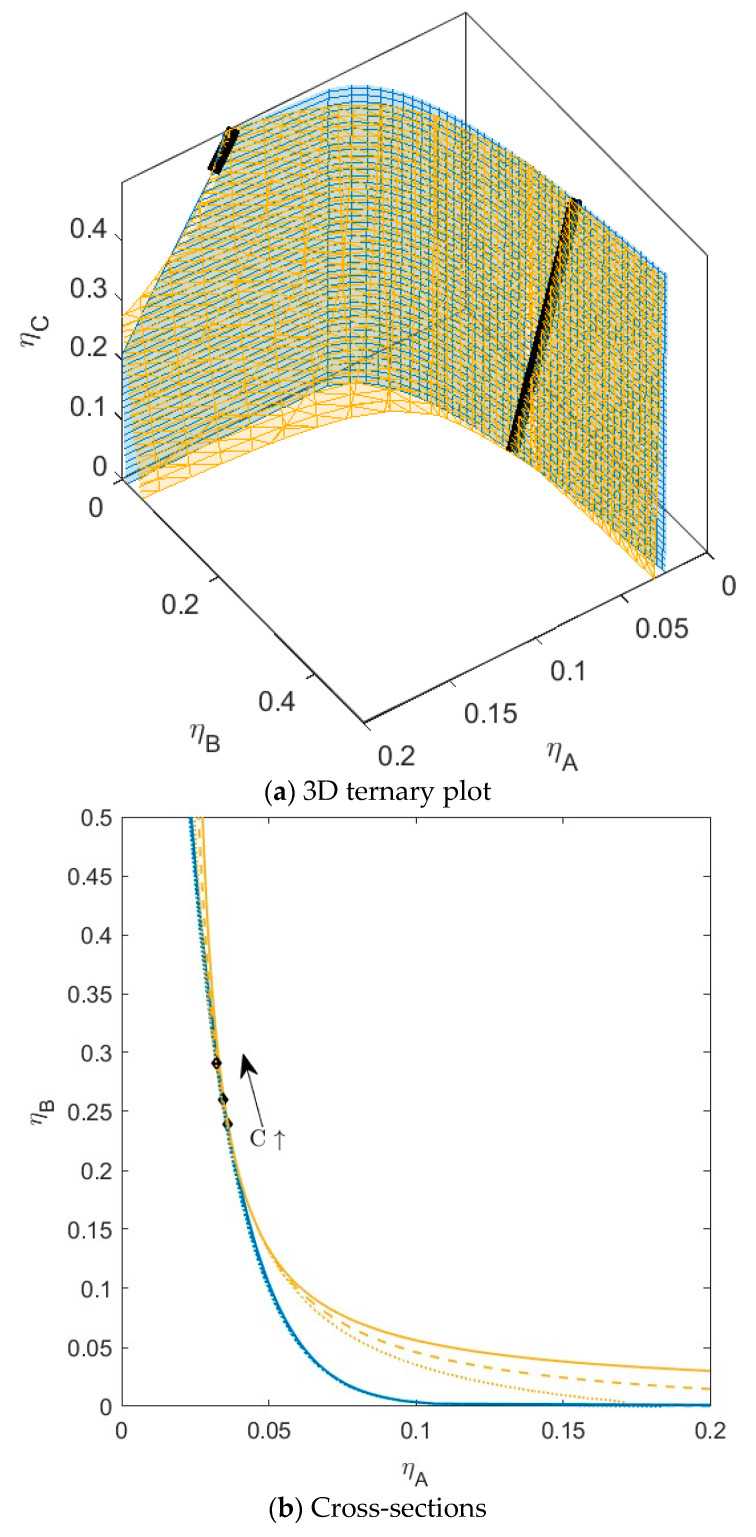
(**a**) Phase diagram for monodisperse ternary (components *A*, *B*, and *C*) non-additive hard sphere mixture with size ratio *q_AB_* = *σ_A_*/*σ_B_* = 1/4 and *q_AC_* = *σ_A_*/*σ_C_* = 1/3, with non-additivity parameters: ∆*_AB_* = 0.1, ∆*_AC_* = 0, and ∆*_BC_* = −0.1, plotted as a function of the partial packing fractions, *η_A_*, *η_B_*, and *η_C_*. The spinodal (yellow surface) and binodal (blue surface) meet each other at the plait point line (black line). (**b**) Phase diagram of component *A* and *B* at specific concentrations of component *C*, yellow lines are the spinodal, blue lines are the binodal, and the black diamonds are the critical points. Full line at concentration *η_C_* = 0.05, dashed line at concentration *η_C_* = 0.15, and dotted line at concentration *η_C_* = 0.30. With increasing concentrations of *C*, the critical point shifts to higher concentrations of *B.*

**Figure 7:**
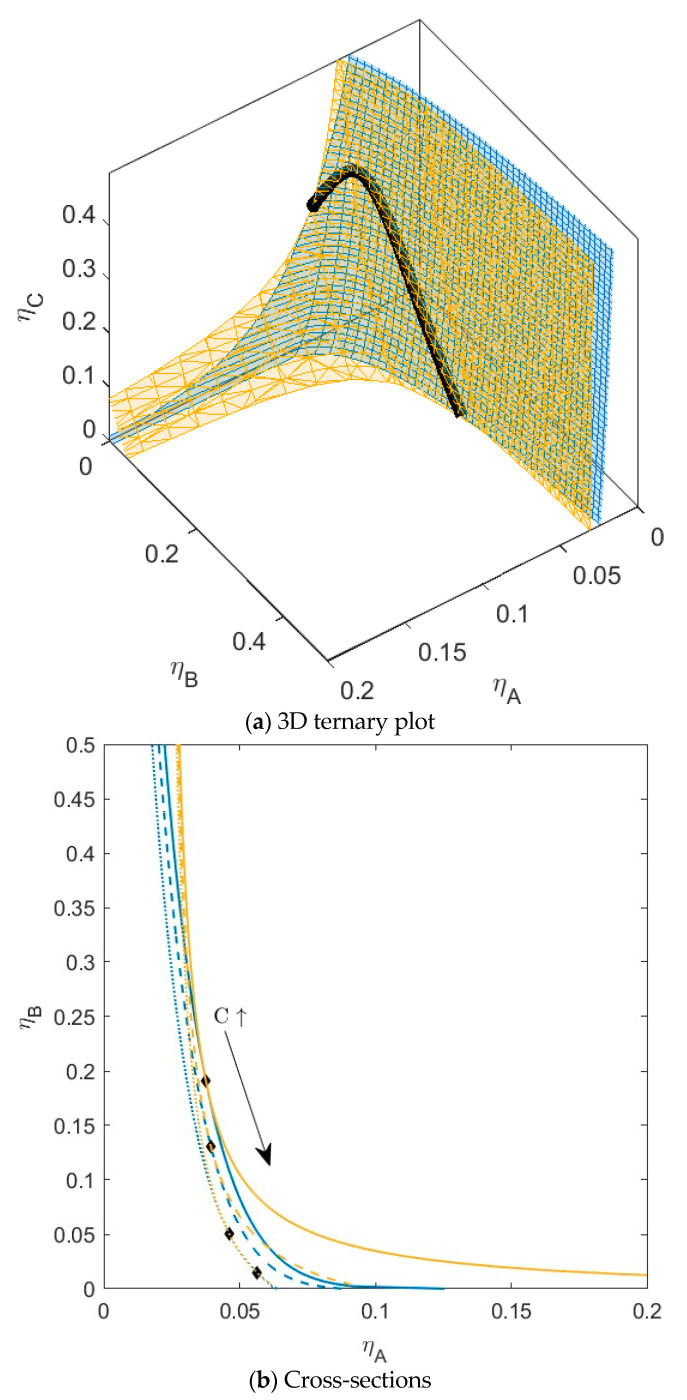
(**a**) Phase diagram for monodisperse ternary (components *A*, *B*, and *C*) non-additive hard sphere mixture with size ratio *q_AB_* = *σ_A_*/*σ_B_* = 1/4 and *q_AC_* = *σ_A_*/*σ_C_* = 1/3, with non-additivity parameters: ∆*_AB_* = 0.1, ∆*_AC_* = 0, and ∆*_BC_* = −0.1, plotted as a function of the partial packing fractions, *η_A_*, *η_B_*, and *η_C_*. The spinodal (yellow surface) and binodal (blue surface) meet each other at the plait point line (black line). (**b**) Cross-sections phase diagram of component *A* and *B* at specific concentrations of component *C*; yellow lines are the spinodal, blue lines are the binodal, and the black diamonds are the critical points. Full line at concentration *η_C_* = 0.05, dashed line at concentration *η_C_* = 0.15, and dotted line at concentration *η_C_* = 0.30. With increasing concentrations of *C*, the critical point shifts to higher concentrations of *B*.

**Figure 8:**
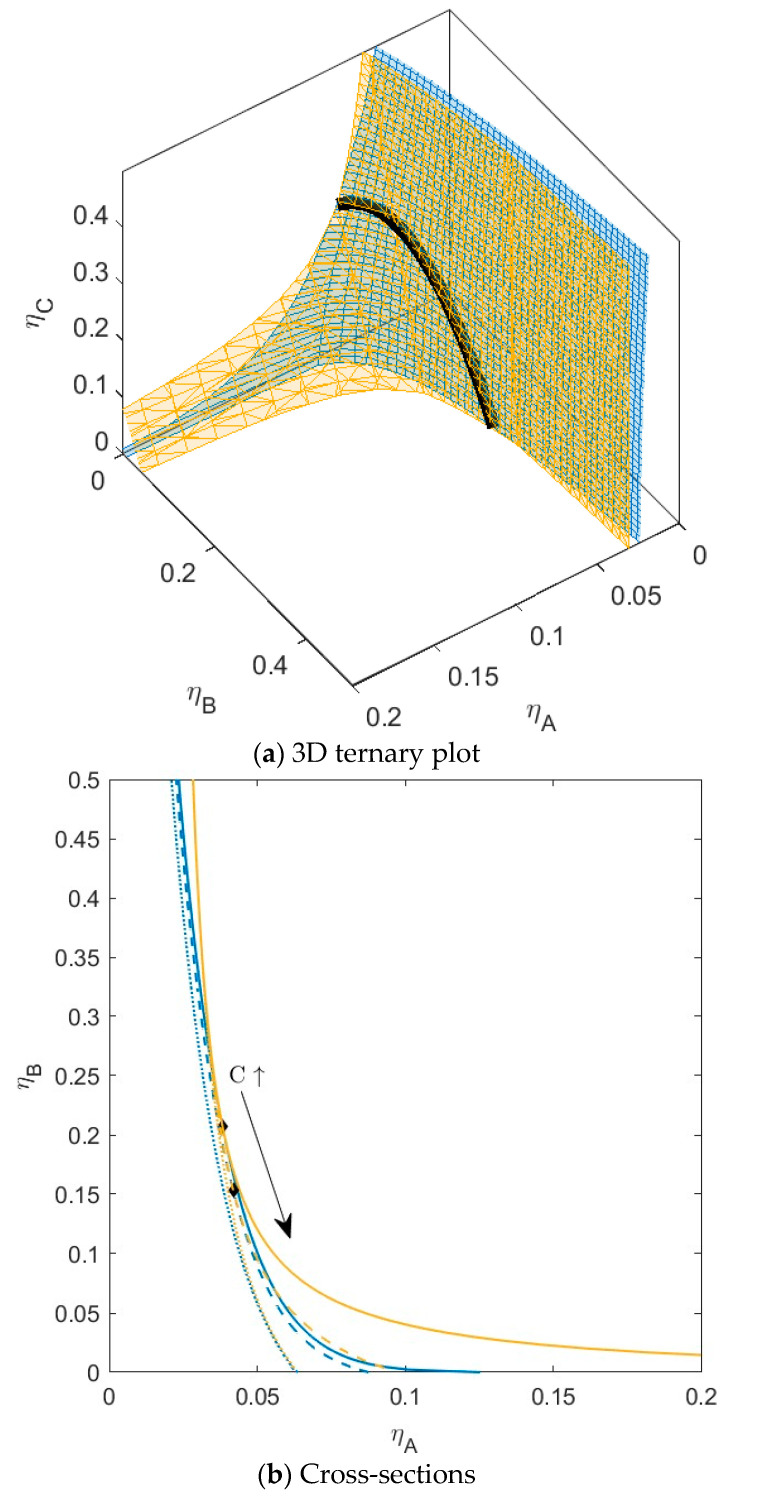
(**a**) Phase diagram for monodisperse ternary (components *A*, *B*, and *C*) non-additive hard sphere mixture with size ratio *q_AB_* = *σ_A_*/*σ_B_* = 1/4 and *q_AC_* = *σ_A_*/*σ_C_* = 1/3, with non-additivity parameters: ∆*_AB_* = 0.1, ∆*_AC_* = 0, and ∆*_BC_* = −0.1, plotted as a function of the partial packing fractions, *η_A_*, *η_B_*, and *η_C_*. The spinodal (yellow surface) and binodal (blue surface) meet each other at the plait point-line (black line). (**b**) Phase diagram of component *A* and *B* at specific concentrations of component *C*; yellow lines are the spinodal, blue lines are the binodal, and the black diamonds are the critical points. Full line at concentration *η_C_* = 0.05, dashed line at concentration *η_C_* = 0.15, and dotted line at concentration *η_C_* = 0.30. With increasing concentration of *C*, the critical point shifts to higher concentration of *B*.

**Figure 9:**
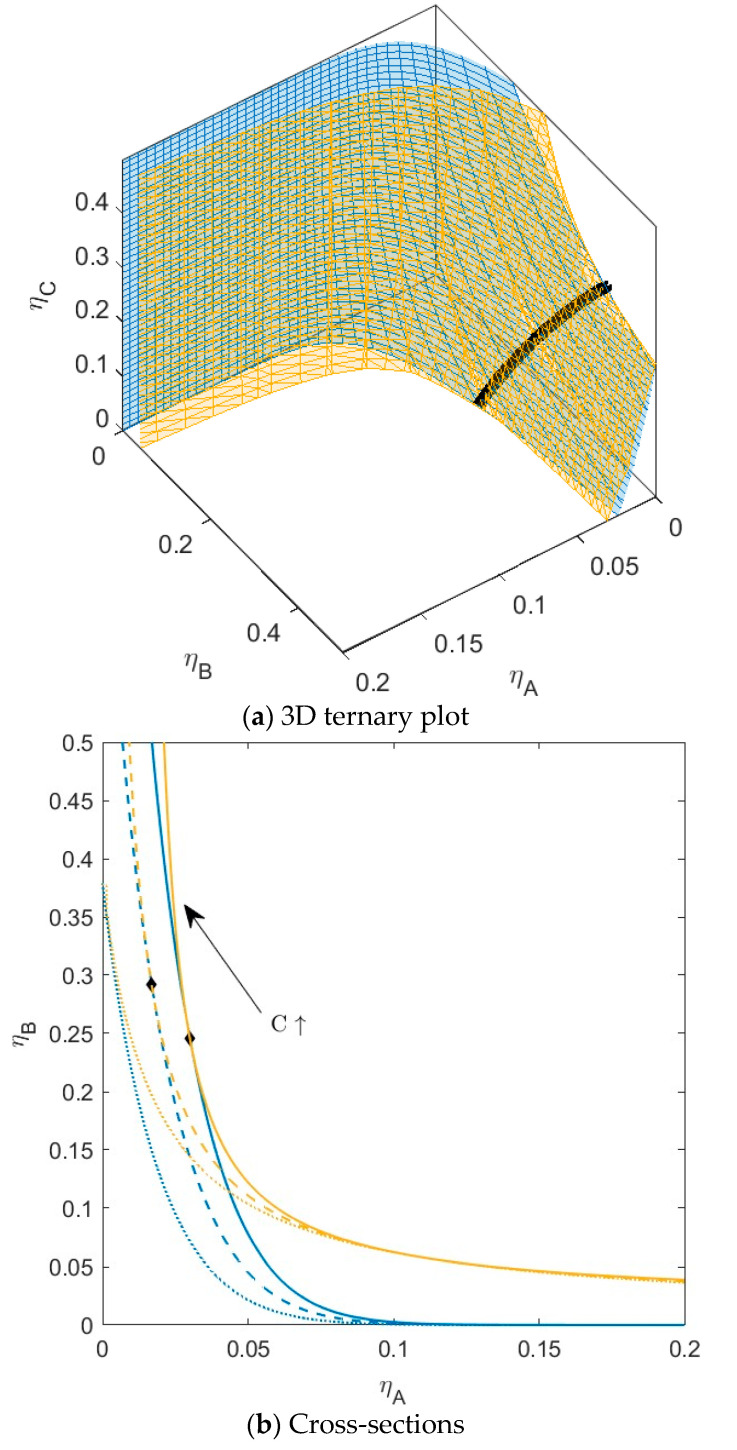
(**a**) Phase diagram for monodisperse ternary (components *A*, *B*, and *C*) non-additive hard sphere mixture with size ratio *q_AB_* = *σ_A_*/*σ_B_* = 1/4 and *q_AC_* = *σ_A_*/*σ_C_* = 1/3, with non-additivity parameters: ∆*_AB_* = 0.1, ∆*_AC_* = 0, and ∆*_BC_* = −0.1, plotted as a function of the partial packing fractions, *η_A_*, *η_B_*, and *η_C_*. The spinodal (yellow surface) and binodal (blue surface) meet each other at the plait point line (black line). (**b**) Phase diagram of components *A* and *B* at specific concentrations of component *C*; yellow lines are the spinodal, blue lines are the binodal, and the black diamonds are the critical points. Full line at concentration *η* = 0.05, dashed line at concentration *η_C_* = 0.15, and dotted line at concentration *η_C_* = 0.30. With increasing concentrations of *C*, the critical point shifts to higher concentrations of *B*.

**Figure 10:**
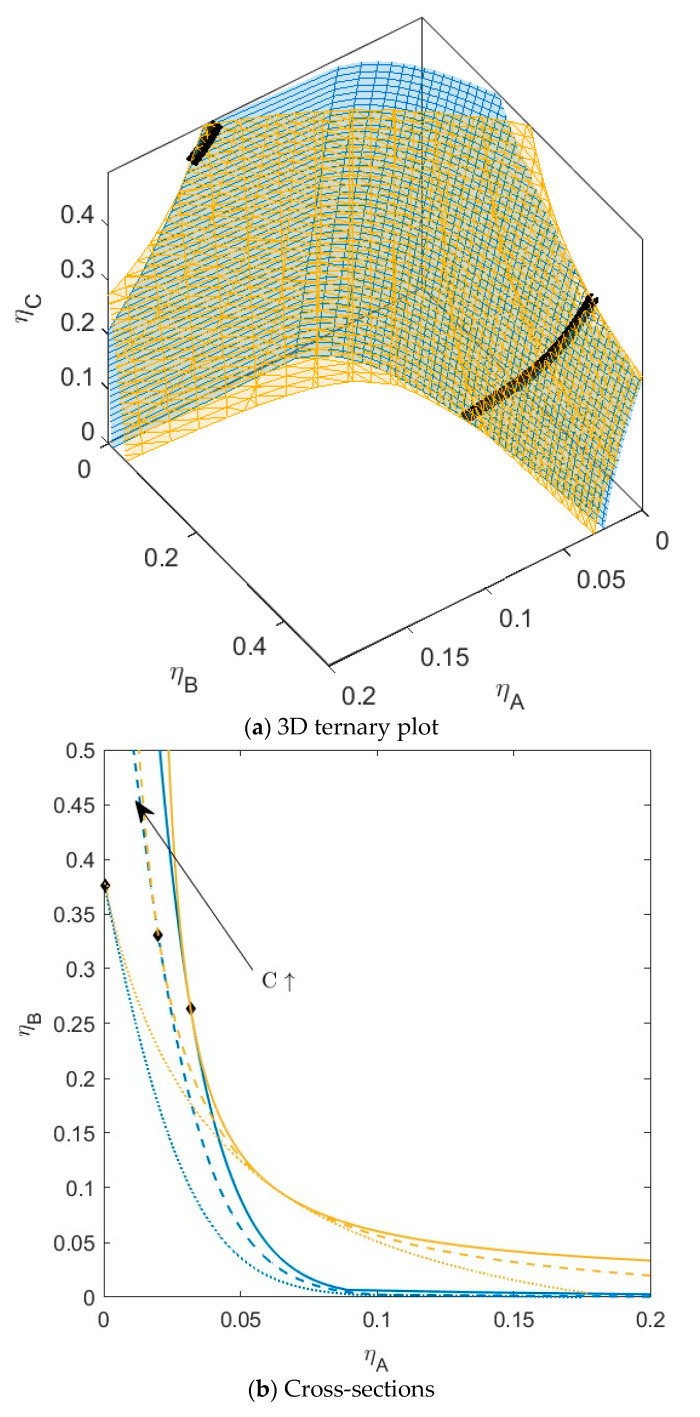
(**a**) Phase diagram for monodisperse ternary (components *A*, *B*, and *C*) non-additive hard sphere mixture with size ratio *q_AB_* = *σ_A_*/*σ_B_* = 1/4 and *q_AC_* = *σ_A_*/*σ_C_* = 1/3, with non-additivity parameters: ∆*_AB_* = 0.1, ∆*_AC_* = 0, and ∆*_BC_* = −0.1, plotted as a function of the partial packing fractions, *η_A_*, *η_B_*, and *η_C_*. The spinodal (yellow surface) and binodal (blue surface) meet each other at the plait point line (black line). (**b**) Phase diagram of components *A* and *B* at specific concentrations of component *C*; yellow lines are the spinodal, blue lines are the binodal, and the black diamonds are the critical points. Full line at concentration = 0.05, dashed line at concentration *η_C_* = 0.15, and dotted line at concentration *η_C_* = 0.30. With increasing concentrations of *C*, the critical point shifts to higher concentrations of *B*.

**Figure 11:**
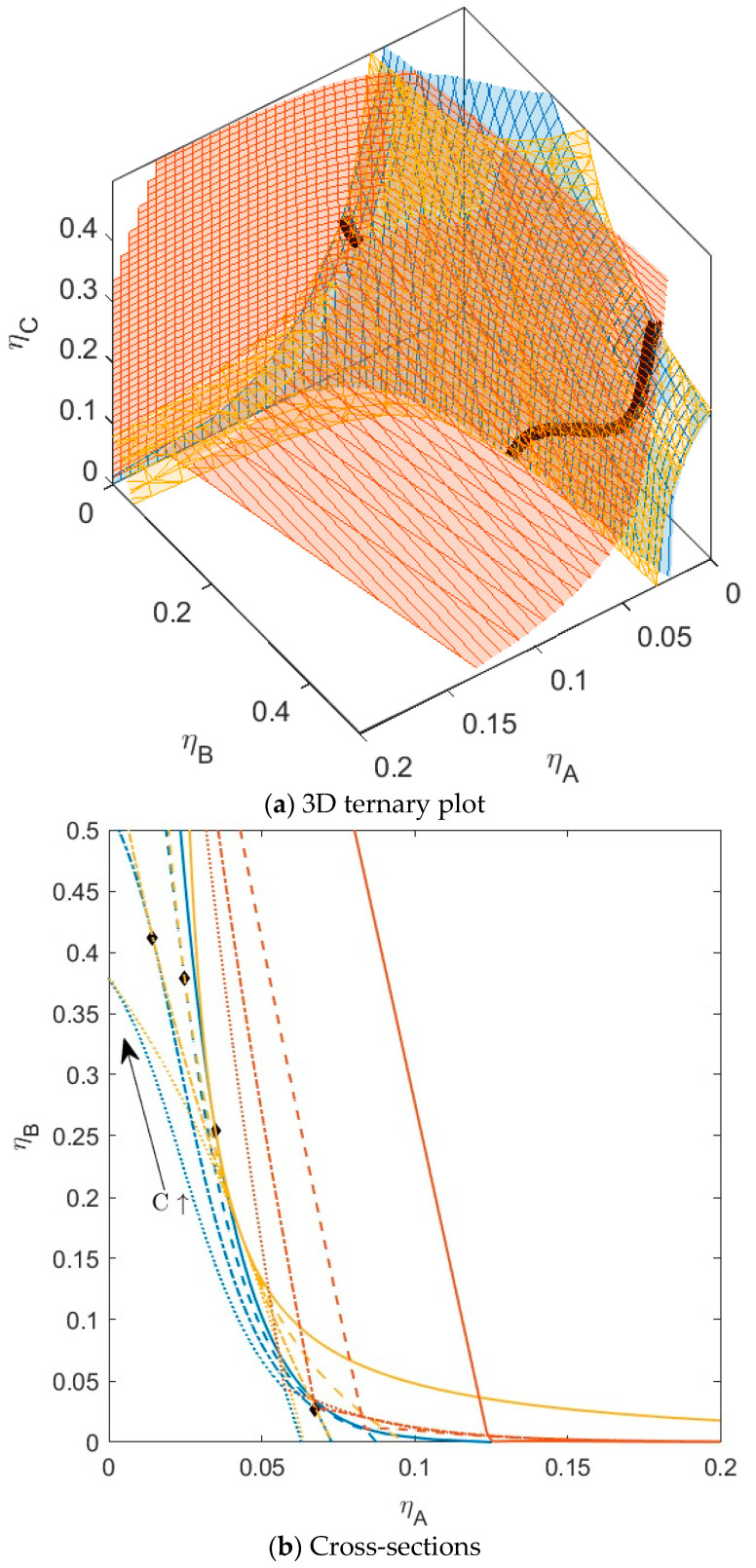
(**a**) Phase diagram for monodisperse ternary (components *A*, *B*, and *C*) non-additive hard sphere mixture with size ratio *q_AB_* = *σ_A_*/*σ_B_* = 1/4 and *q_AC_* = *σ_A_*/*σ_C_* = 1/3, with non- additivity parameters: ∆*_AB_* = 0.1, ∆*_AC_* = 0.1, and ∆*_BC_* = 0.1, plotted as a function of the partial packing fractions, *η_A_*, *η_B_*, and *η_C_*. The spinodal (yellow surface) and binodal (blue surface) meet each other at the plait point line (black line). The mixture also has a three-phase boundary (red surface). (**b**) Phase diagram of components *A* and *B* at specific concentrations of component *C*; yellow lines are the spinodal, blue lines are the binodal, and the black diamonds are the critical points. Full line at concentration *η_C_* = 0.05, dashed line at concentration *η_C_* = 0.15, and dotted line at concentration *η_C_* = 0.227, and dotted line at concentration *η_C_* = 0.30.

We define a specific binary mixture of components *A* and *B*, to which we add a third component *C*. The size ratio between the small sphere *A* and the larger sphere *B* is *q_AB_* = *σ_A_*/*σ_B_* = 1/4, and the non-additivity parameter ∆*_AB_* = 0.1. The critical point for the binary mixture *AB* is at *η_crit_* = (0.037, 0.229). To this mixture, we add a component *C*, with size ratio *q_AC_* = *σ_A_*/*σ_C_* = 1/3. The size of *C* is therefore in-between the sizes of *A* and *B*. We vary the non-additivity parameters (∆*_AC_* and ∆*_BC_*) between components *A* and *C*, and *B* and *C*, respectively, from ∆ = −0.1 to ∆ = 0.1, simulating a range of thermodynamic compatibility and incompatibility between the three components. The phase diagram of this binary mixture of *A* and *B* can be found in Figure 2. We calculate the plait points, the phase separation boundary, and the spinodal of the various ternary mixtures (see Figure 3, Figure 4, Figure 5, Figure 6, Figure 7, Figure 8, Figure 9, Figure 10 and Figure 11).

Mixtures are able to demix when the stability matrix (Equation (9)) has a negative eigenvalue (in our case, we look at total concentration *η <* 0.5). An easy way to check for the possibility of phase separation for binary mixtures is by using the following parameter [29]: $B_{crit}=B_{12}^{2}/B_{11}B_{22}$, where $B_{crit}>1$ implies phase separation possibility. The parameter is a measure for the compatibility between the two components. The higher the value, the more incompatible the components are. The binary mixture *AB* has a *B_crit_* of 6.76. For mixtures with more than two components, we have to find another criterion to check for the possibility of two- or three-phase separation. We will therefore first investigate a number of different mixtures depending on the interactions in the binary sub-mixtures. We start with mixtures for which the component *C* does not show any phase separation at concentrations *η <* 0.5 with *A* or *B*. In this case, *C* shows thermodynamic compatibility with both *A* and *B*. In the next section, *C* is incompatible with *A* or *B*. The binary mixture *AC* or *BC* will phase separate and have a critical point at concentrations *η <* 0.5. In the third section, *C* is incompatible with both *A* and *B*. The binary mixtures *AC* and *BC* both have a critical point at concentrations *η <* 0.5. For this type of mixture, we hypothesize that it becomes possible that the mixture will phase separate into more than two phases, inspired by the hypothesis put forward by the work of Mace et al. [10], who report on multiphase systems containing polymers and surfactants.

For the composition of the various phases, we refer also to Figures S1–S9 in Supplementary.

### 3.1. C Compatible with Both A and B

In the first mixture (Figure 3), we set the non-additivity parameter for both the interactions with *C* to −0.1 (∆*_AC_* = −0.1 and ∆*_BC_* = −0.1). In this case, the sub-mixture *BC* will not demix at any concentration, because *B_critBC_ <* 1 (see Table 1); the size difference between *B* and *C* combined with the negative non-additivity is not enough for phase separation. The sub-mixture *AC* can theoretically phase separate (*B_critAC_ >* 1); however, the critical point is at unattainably high concentrations (*η_crit_ >* 1). For the ternary mixture, we see that the addition of the third component *C* to the binary mixture *AB* has little effect on the phase behavior of the two components. With increasing concentrations of *C*, the plait points shift to slightly higher concentrations of *B*, and the spinodal shifts towards lower concentrations of *B* at higher concentrations of *A*. There is no difference in the position of the binodal. With increasing concentration of *C*, the two phases will have the same volume fraction (*α*) and the same demixing of *A* and *B*, independent of *C*. See also Figure S1 (Supplementary). Component *C* is present in both phases with concentrations similar to the concentration in the parent phase. For this system, the tie lines show very little rotation, so they are relatively parallel to the *xy*-plane.

For the next mixture (Figure 4), we kept the interaction between *A* and *C* the same, but we increased the non-additivity parameter between *B* and *C* to hard sphere interaction (∆*_AC_* = −0.1 and ∆*_BC_* = 0). Now, it is theoretically possible for *B* and *C* to phase separate (*B_crit__BC_ >* 1) (see Table 1); however, just as for *A* and *C*, the critical point is at unattainably high concentrations. For this mixture, we see an increased shift in the position of the plait points, again towards higher concentrations for *B* with an increase in the concentration of *C*. For this mixture, the binodal also starts to shift towards slightly lower concentrations of *A* at higher concentrations of *C*.

The fact that the plait points shift to different concentrations of *A* and *B* with the addition of the third component *C* is an indication that the volumes of both phases will be different for specific mixtures of *A* and *B* with the addition of *C*. Not only will the volumes change, but the preferential fractionation of the components in both phases will also be different. The more the plait points shift to different concentrations, the more the tie lines between phases will show rotation with the addition of the third component. This can be seen in Figure S2 (Supplementary), where component *C* has a preference of going to the top phase (phase enriched in *A*), and the volume fraction (a) of the bottom phase (phase enriched in *B*) decreases slightly with an increased addition of *C*. The tie lines in this system start to show some rotation.

### 3.2. C Compatible with A or B and Incompatible with the Other One

For the mixture in Figure 5, we switched the non-additivity parameters from the previous mixture to ∆*_AC_* = 0 and ∆*_BC_* = −0.1. The sub-mixture *AC* can phase separate at concentrations *η <* 0.5, but the sub-mixture *BC* cannot demix at all (see also Table 1). For this three-component mixture, we see that the binodal surface at *η_B_* = 0 intersects with the *xz*-plane, indicating phase separation between the binary mixture of *A* and *C*. The mixture yields a plait point line that has broken up into two sections. These sections meet at very high concentrations of *C*. In the cross-sections (Figure 5b), we see that the plait points, as well as the spinodal at high concentrations of *A*, shift towards lower concentrations of *B* with increasing concentration of *C*. There is little difference in the phase separation boundary for an increasing concentration of *C*; however, the tie lines do rotate, one end being at slightly lower concentrations of *C*, the other end at higher concentrations of *C* compared to the parent phase.

In the mixture in Figure 6 the interactions between both *A* and *C* and *B* and *C* are additive hard sphere interactions (∆*_AC_* = ∆*_BC_* = 0). The sub-mixture *AC* can phase separate at concentrations *η <* 0.5, but the sub-mixture *BC* cannot demix at this low concentration, because the size difference between *B* and *C* is not large enough (see also Table 1). For this three-component mixture, we see that the binodal surface at *η_B_* = 0 intersects with the *xz*- plane, indicating phase separation between *A* and *C*, just as in the previous mixture. The mixture has two sections of plait point line, again just as the previous mixture; here, however, the plait point line first bends away from the short section, to meet the other section at very high concentrations of *C*. In the cross-sections (Figure 6b), we see that the plait points shift towards higher concentrations of *B* with increasing concentrations of *C*, and the spinodal at high concentrations of *A* shifts towards lower concentrations of *B*.

For the next mixture (Figure 7), the non-additivity parameters were set to ∆*_AC_* = 0.1 and ∆*_BC_* = −0.1. Due to the increased incompatibility between *A* and *C*, compared to Figure 5, the sections of plait pint line meet each other at lower concentrations. In the cross-sections (Figure 7b), the cross-section at the highest concentration of *C* intersects this line twice, leading to two plaint points at *η_C_* = 0.30. Because component *C* had higher compatibility with *B* compared to *A*, this component now preferentially goes to the bottom phase upon phase separation. With an increased concentration of *C*, the volume fraction (a) of the bottom phase increases as well (see also Figure S5, bottom), indicating the more pronounced rotation of the tie lines connecting the child phases with the parent phase in a straight line.

With increased incompatibility between *B* and *C* (∆*_AC_* = 0.1 and ∆*_BC_* = 0), the sections of plait point line meet each other at even lower concentrations (Figure 8). In the cross-sections (Figure 8b), the cross-section at the highest concentration of *C* does not have a plait point anymore. Component *C* still has more affinity with component *B* and prefers the bottom phase upon phase separation (Figure S6, bottom); however, due to the increased incompatibility between *B* and *C*, the volume fraction (*α*) of the bottom phase increases more upon phase separation compared to the previous mixture.

In the mixture in Figure 9, *A* and *C* have a negative non-additivity parameter, and *B* and *C* have a positive non-additivity parameter (∆*_AC_* = −0.1 and ∆*_BC_* = 0.1). The sub- mixture *AC* does not have a critical point at physically relevant concentrations to phase separate; however, the sub-mixture *BC* does phase separate. For the ternary mixture, the plait points shift towards higher concentrations of *B* with increasing concentration of *C*, until the critical point of the sub-mixture *BC* is reached. With decreasing concentration of *A*, the binodal and spinodal bend towards the *yz*-plane, until the surfaces meet with the binodal and spinodal of the binary *BC* mixture. In the cross-section (Figure 9b), this can be seen as with increasing concentrations of *C*, the binodal shifts towards lower concentrations of *A*. Component *C* is most compatible with component *A*; therefore, upon phase separation, component *C* prefers the top phase. With increased concentrations of *C*, the volume fraction (*α*) of the top phase increases (see also Figure S7).

### 3.3. C Incompatible with Both A and B

The three sub-mixtures of the mixture in Figure 10 (with ∆*_AC_* = 0 and ∆*_BC_* = 0.1) all show phase separation, as can be seen from both the binodal and the spinodal surfaces shifting towards the *xz*-plane and *yz*-plane (see also Table 1). We hypothesized that this type of mixture can demix into three phases; however, as can be seen in the graph, this mixture does not show three-phase separation. The lowest concentration of the three-phase boundary is at concentrations *η > 1* and therefore unattainable. There are again two sections of plait point lines for this mixture, just as in some of the mixtures in the previous section. These two sections of plain point lines do not meet each other, not even at very high concentrations of component *C*. In the cross-sections shown in Figure 10b, we see that with increasing concentration of component *C*, the plait point shifts to lower concentrations of *A* and higher concentrations of *B*.

The three sub-mixtures of the mixture in Figure 11 (with ∆*_AC_* = 0.1 and ∆*_BC_* = 0.1) all show phase separation. For this mixture, both the binodal and the spinodal surfaces shift towards the *xz*-plane and *yz*-plane as well. In this case, the incompatibility between all three components is large enough for the mixture to demix into three phases at physically relevant concentrations. The lowest concentration of the three-phase boundary is at *η_crit_* = (0.067, 0.028, 0.227). This point is on the spinodal, the plait point line, and also the binodal. This is a special plait point, where all three phases become indistinguishable. Small deviations in one of the concentrations of the three components will result in the formation of one homogeneous phase, two phases (depending on the perturbation, these phases are enriched in different components), or three phases. There are again two sections of plait point lines for this mixture, just as in some of the mixtures in the previous section. These two sections of plait point lines do not meet each other, not even at very high concentrations of *C*, since one section terminates at the three-phase boundary. The two-phase boundary has a bend where the surface meets with the three-phase boundary. Depending on the concentrations of *A*, *B*, and *C*, the two phases will have very different fractionation.

### 3.4. Fractionation

**Figure 12:**
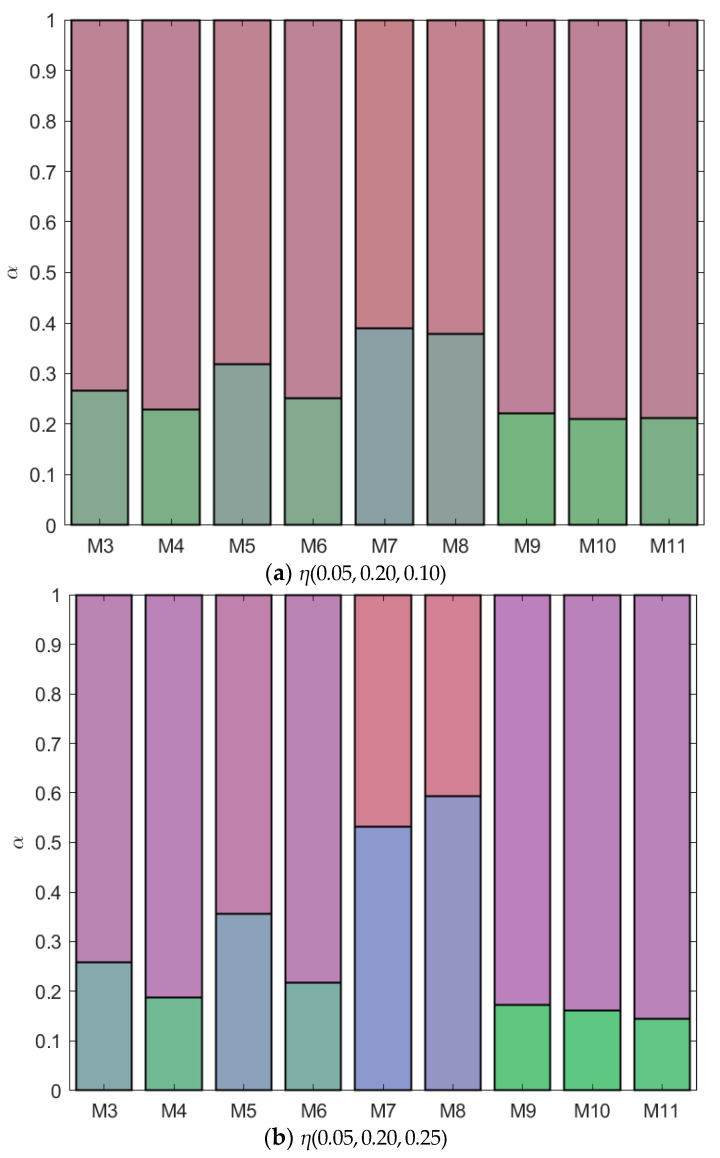
Fractionation of monodisperse ternary (components *A*, *B*, and *C*) non-additive hard sphere mixtures with size ratio *q_AB_* = *σ_A_*/*σ_B_* = 1/4 and *q_AC_* = *σ_A_*/*σ_C_* = 1/3, with non-additivity parameters: ∆*_AB_* = 0.1, ∆*_AC_* and ∆*_BC_* varying from −0.1 to 0.1. Label referring to Figure 3, Figure 4, Figure 5, Figure 6, Figure 7, Figure 8, Figure 9, Figure 10 and Figure 11, at fixed parent phase; *A* is red, *B* is green, and *C* is blue; table with concentrations in Supplementary Materials.

In the previous sections, we saw the phase diagrams of nine different mixtures for which we altered the interactions between a third component *C* and two components *A* and *B* that phase separate. In this section, we will qualitatively compare the phase behavior of the different mixtures at specific parent concentrations of the different components to obtain more insight into the phase separation dynamics. In all the fractionation figures, each component has a different color: component *A* is red, component *B* is green, and component *C* is blue (see Supplementary Materials for a table). Depending on the concentration of each component in each phase, the color in the figure will be a combination of the different colors. In Figure 12a, the parent phase has a concentration of *η*(0.05, 0.20, 0.10). For all mixtures, component *A*, being the smallest component in the mixture, preferentially goes to the top phase, while component *B*, the largest component in the system, remains at the bottom. The volume fraction of the bottom phase (*α*) is largest for the mixtures from Figure 5, Figure 7, and Figure 8. For these mixtures, *A* and *C* were more incompatible than *B* and *C*, as can be seen in Table 1 from their *B_crit_* values. Component *C* preferentially goes to the bottom phase (more blue in bottom phase compared to top phase) at this parent concentration, and the volume fraction of the bottom phase correspondingly increases.

Looking at much higher concentrations of *C* (*η*(0.05, 0.20, 0.25), Figure 12b), we see that for most mixtures component *C* prefers the top phase, but for the two mixtures with the highest incompatibility between *A* and *C* and low to moderate incompatibility between *B* and *C* (the mixtures from Figure 7 and Figure 8), component *C* clearly moves to the bottom phase. The higher the concentration of *C* in a phase, the more the volume fraction *α* shifts towards this phase. The bottom phase of the mixture from Figure 8 is the largest, whilst the bottom phase of the mixture from Figure 11 is the smallest.

**Figure 13:**
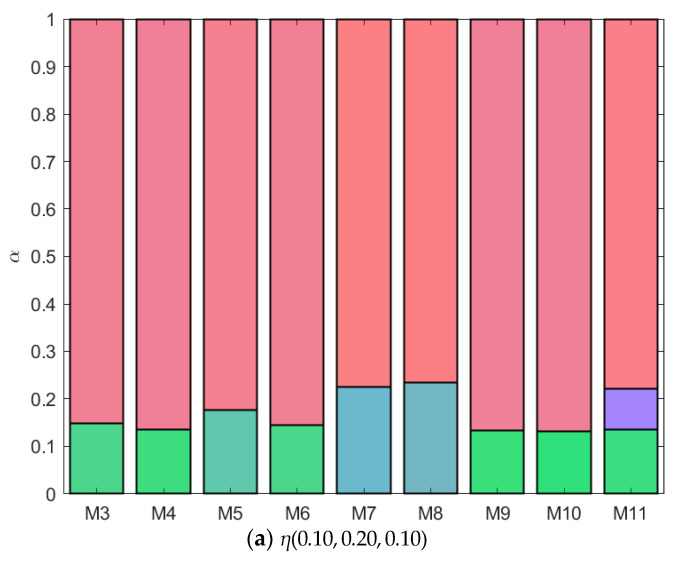

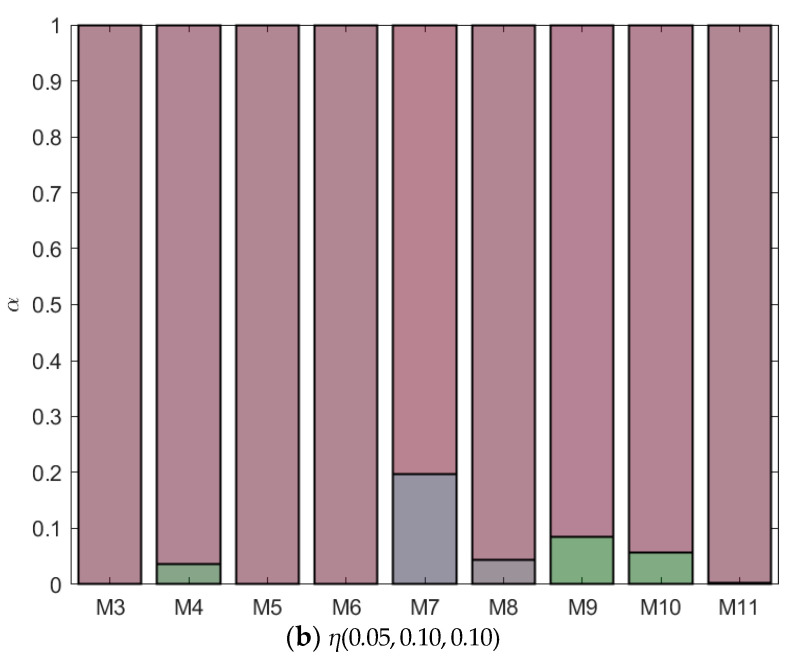
Fractionation of monodisperse ternary (components *A*, *B*, and *C*) non-additive hard sphere mixtures with size ratio *q_AB_* = *σ_A_*/*σ_B_* = 1/4 and *q_AC_* = *σ_A_*/*σ_C_* = 1/3, with non-additivity parameters: ∆*AB* = 0.1, ∆*_AC_* and ∆*_BC_* varying from −0.1 to 0.1. Label referring to Figure 3, Figure 4, Figure 5, Figure 6, Figure 7, Figure 8, Figure 9, Figure 10 and Figure 11, at fixed parent phase; *A* is red, *B* is green, and *C* is blue; table with concentrations in Supplementary Materials.

When increasing the concentration of component *A* on the other hand (*η*(0.10, 0.20, 0.10), Figure 13a), we see that for the mixture from Figure 11, a third phase appears. At the higher concentration of *A*, component *C* demixes from the top phase, forming the new middle phase. Finally, we look at a parent concentration with a lower amount of *B* (*η*(0.05, 0.10, 0.10), Figure 13b). At this concentration, the mixture *AB* does not show phase separation yet. However, due to the addition of component *C*, some of the mixtures demix at this parent concentration. This is especially true for the mixtures with more compatibility between *B* and *C* and higher incompatibility between *A* and *C*, the mixtures from Figure 7 and Figure 8. The mixture from Figure 11 has the same incompatibility between *A* and *C*; however, due to the increased incompatibility between *B* and *C*, the mixture remains stabilized. When a mixture demixes into two or more phases, the concentration of one or more components is higher in one of the phases compared to the other, while each of the phases has a smaller volume compared to the parent volume. Systems with less incompatibility among some of the components tend to demix at slightly lower concentration compared to systems with high incompatibility. This was also shown experimentally by Johansson and Walter [30] for the system polyethylene glycol and dextran, where a third polymer added to a dilute one-phase mixture of polyethylene glycol and dextran caused the system to demix into two phases. At low concentrations, this third polymer distributes between the two phases.

## 4. Three-Phase Systems

**Figure 14:**
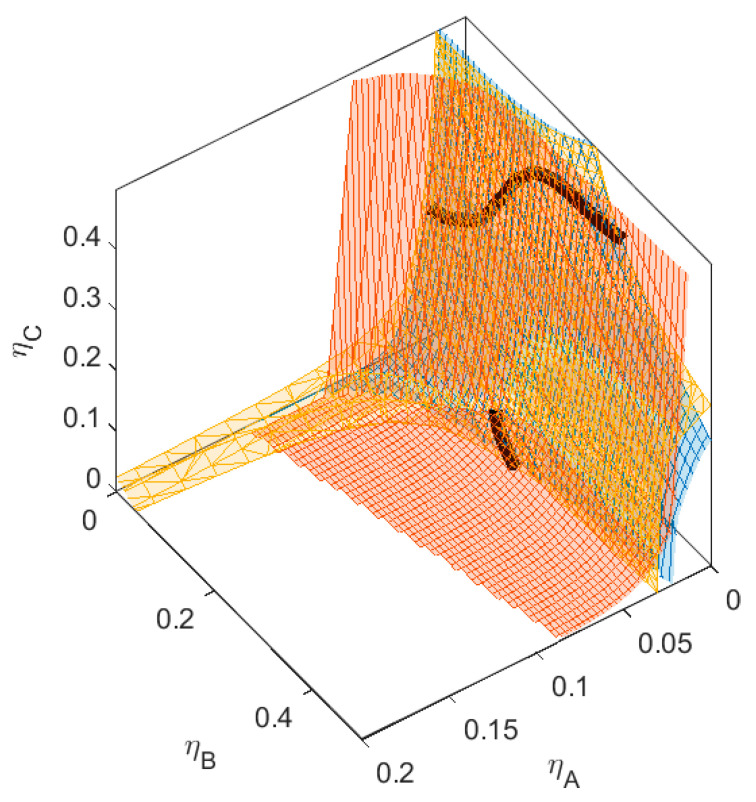
Phase diagram for monodisperse ternary (components *A*, *B*, and *C*) non-additive hard sphere mixture with size ratio *q_AB_* = *σ_A_*/*σ_B_* = 1/4 and *q_AC_* = *σ_A_*/*σ_C_* = 1/5, with non-additivity parameters: ∆*_AB_* = 0.1, ∆*_AC_* = 0.1, and ∆*_BC_* = 0.1, plotted as a function of the partial packing fractions, *η_A_*, *η_B_*, and *η_C_*. The spinodal (yellow surface) and binodal (blue surface) meet each other at the plait point line (black line). The mixture also has a three-phase boundary (red surface).

In the previous sections, we saw one three-phase system. We changed the size of *C* and the non-additive interaction parameters ∆*_AC_* and ∆*_BC_* to investigate the dynamics in three- phase systems. First, we changed the size ratio between *A* and *C*. For the mixture in Figure 14, the size ratio is *q_AC_* = 1/5, and the non-additive interactions between *C* and the other components are ∆*_AC_* = 0.1 and ∆*_BC_* = 0.1 (the same as for Figure 11). When looking at the *B_crit_* (see Table 2), we see that this change in size resulted in more repulsive interaction between *A* and *C*, while *B_critBC_*remains more or less the same. This can also be seen in the concentration of the critical points of their respective binary mixtures (e.g., for the mixture *BC*, the total volume fraction at the critical point remains more or less the same); however, their respective fractions are flipped due to their flip in size ratio. Compared to Figure 11, the three-phase boundary is rotated. The critical point of the three-phase boundary is at *η*(0.035, 0.188, 0.052). Since component *C* is now the largest component in the system (and therefore the heaviest of the three, assuming the density of the components is larger than the density of the solution), this component preferentially goes to the bottom phase, component *B* preferentially goes to the middle phase, and component *A* is most abundant in the top phase. When we navigate through the phase diagram on a straight line with increasing component A while keeping the concentration of components B and C the same, the system first demixes into a large top phase enriched in component A and B and a small bottom phase enriched in component C. This is due to the higher incompatibility of C with A compared to the incompatibility between A and B. When more of component A is added, component B demixes from the top phase, forming the middle phase.

**Figure 15:**
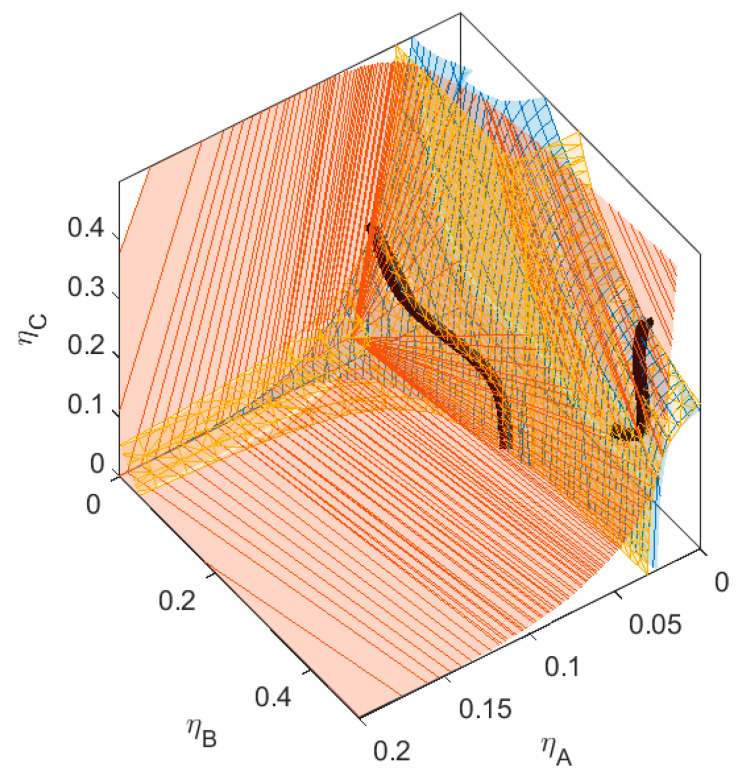
Phase diagram for monodisperse ternary (components *A*, *B*, and *C*) non-additive hard sphere mixture with size ratio *q_AB_* = *σ_A_*/*σ_B_* = 1/4 and *q_AC_* = *σ_A_*/*σ_C_* = 1/3, with non-additivity parameters: ∆*_AB_* = 0.1, ∆*_AC_* = 0.15, and ∆*_BC_* = 0.1, plotted as a function of the partial packing fractions, *η_A_*, *η_B_*, and *η_C_*. The spinodal (yellow surface) and binodal (blue surface) meet each other at the plait point line (black line). The mixture also has a three-phase boundary (red surface).

In Figure 15, we again used the same size of *C* as in the first series of mixtures (*q_AC_* = 1/3), and we increased the incompatibility between *A* and *C*. Compared to Figure 11 and Figure 14, the three-phase boundary is rotated. The critical point of the three-phase system is at *η*(0.027, 0.412, 0.169). When gradually increasing the concentration of component *C*, the two-phase system of one phase enriched in *A* (the top phase), and the other phase enriched in *B*. When reaching the three-phase boundary, component *C* demixes from the top phase, forming the middle phase, which is enriched in component *C*.

Experimental three-phase systems of, for example, the colloids dextran, ficoll, and polyethylene-glycol [8] and dextran, polyethylene-glycol, and gelatin (unpublished work) show similar phase behavior. Depending on the concentrations of each component, the system will form one phase or demix into two or three phases. Each phase enriched in one of the components.

## 5. Conclusions

The addition of a third component *C* to a binary mixture *AB* that demixes has a different impact depending on the pairwise compatibility between the third components and the two other components. Depending on the pairwise interactions for the three components, the volume fractions and compositions of the three phases are altered. If the third component is compatible with both components, the phase boundary and spinodal are nearly vertical surfaces. The third component therefore does not decrease the incompatibility between the components *A* and *B*. The component *C* is present in both phases in the same amount, and the volume fraction between the phases is not altered by the addition of the third component. The plait point line of the mixture will shift to lower or higher concentrations of the components *A* and *B* with increasing concentration of *C* depending on the interaction of *C*. The sum of the volume fractions of *A* and *B* is, however, always equal or lower compared to the sum of the volume fractions at the critical point of the binary mixture.

When *C* is incompatible with one of the components *A* or *B*, the binodal and spinodal will bend towards one of the planes (either the *xz*-plane or *yz*-plane) of the phase diagram. The plait point line forms a curve that connects the critical points of the binary mixtures that demix. When all three components are incompatible, it becomes possible for the mixture to demix into three phases. The lowest concentration of the three-phase boundary also lays on the spinodal, and for some of the mixtures on the two-phase boundary as well. For these mixtures, small perturbations in the concentrations of one of the components at this critical point will result in one homogeneous phase, two phases, or three phases. Depending on the different pairwise interactions between the components, the three-phase boundary has a different shape and rotation.

## Figures and Tables

**Table 1:** Virial coefficient ratios (*B_crit_*) for binary mixtures with *C* depending on the non-additivity parameter ∆ and critical point for binary mixtures that phase separate (the interaction between *A* and *B* remains the same for all mixtures).

Mixture	∆*_AC_*	*B* *_critAC_*	*η* *_critAC_*	∆*_BC_*	*B* *_critBC_*	*η* *_critBC_*
3	−0.10	1.26		−0.10	0.57	
4	−0.10	1.26		0	1.06	
5	0	2.37	(0.147, 0.442)	−0.10	0.57	
6	0	2.37	(0.147, 0.442)	0	1.06	
7	0.10	4.20	(0.069, 0.253)	−0.10	0.57	
8	0.10	4.20	(0.069, 0.253)	0	1.06	
9	−0.10	1.26		0.10	1.88	(0.386, 0.295)
10	0	2.37	(0.147, 0.442)	0.10	1.88	(0.386, 0.295)
11	0.10	4.20	(0.069, 0.253)	0.10	1.88	(0.386, 0.295)

**Table 2:** Virial coefficient ratios (*B_crit_*) for binary mixtures with *C* depending on the non-additivity parameter ∆ and size ratio of *A* and *C* and critical point for binary mixtures that phase separate (the interaction between *A* and *B* remains the same for all mixtures). All systems show three-phase separation at concentrations *η <* 0.5.

Mixture	*q_AC_*	∆*_AC_*	*B_critAC_*	*η_critAC_*	∆*_BC_*	*B_critBC_*	*η_critBC_*
11	1/3	0.10	4.20	(0.069, 0.253)	0.10	1.88	(0.386, 0.295)
14	1/5	0.10	10.33	(0.022, 0.219)	0.10	1.84	(0.318, 0.391)
15	1/3	0.15	5.48	(0.052, 0.208)	0.10	1.88	(0.386, 0.295)

## Data Availability

Data details are available upon request.

## Supplementary Materials

The following supporting information can be downloaded at: https://www.mdpi.com/article/10.3390/molecules28237817/s1, Figure S1: Fractionation of monodisperse ternary (component *A*, *B*, and *C*) non-additive hard sphere mixture with size ratio *q_AB_* = *σ_A_/σ_B_* = 1*/*4 and *q_AC_* = *σ_A_/σ_C_* = 1*/*3, with non-additivity parameters: ∆*_AB_* = 0.1, ∆*_AC_* = −0.1, and ∆*_BC_* = −0.1, fixed parent phase: *η*(0.05, 0.20, 0.10), adjusting *A*, *B*, resp. *C* with *η* = 0.01,  *A* is red,  *B* is green,  *C* is blue. Figure S2: Fractionation of monodisperse ternary (component *A*, *B*, and *C*) non-additive hard sphere mixture with size ratio *q_AB_* = *σ_A_/σ_B_* = 1*/*4 and *q_AC_* = *σ_A_/σ_C_* = 1*/*3, with non-additivity parameters: ∆*_AB_* = 0.1, ∆*_AC_* = −0.1, and ∆*_BC_* = 0, fixed parent phase: *η*(0.05, 0.20, 0.10),  *A* is red,  *B* is green,  *C* is blue. Figure S3: Fractionation of monodisperse ternary (component *A*, *B*, and *C*) non-additive hard sphere mixture with size ratio *q_AB_* = *σ_A_/σ_B_* = 1*/*4 and *q_AC_* = *σ_A_/σ_C_* = 1*/*3, with non-additivity parameters: ∆*_AB_* = 0.1, ∆*_AC_* = 0, and ∆*_BC_* = −0.1, fixed parent phase: *η*(0.05, 0.20, 0.10),  *A* is red,  *B* is green,  *C* is blue. Figure S4: Fractionation of monodisperse ternary (component A, B, and C) non-additive hard sphere mixture with size ratio *q_AB_* = *σ_A_/σ_B_* = 1*/*4 and *q_AC_* = *σ_A_/σ_C_* = 1*/*3, with non-additivity parameters: ∆*_AB_* = 0.1, ∆*_AC_* = 0, and ∆*_BC_* = 0, fixed parent phase: *η*(0.05, 0.20, 0.10), adjusting *A*, *B*, resp. *C* with *η* = 0.01,  *A* is red,  *B* is green, and  *C* is blue. Figure S5: Fractionation of monodisperse ternary (component A, B, and C) non-additive hard sphere mixture with size ratio *q_AB_* = *σ_A_/σ_B_* = 1/4 and *q_AC_ = σ_A_/σ_C_* = 1/3, with non-additivity parameters: ∆*_AB_* = 0.1, ∆*_AC_* = 0.1, and ∆*_BC_* = −0.1, fixed parent phase: *η*(0.05, 0.20, 0.10), adjusting A, B, resp. C with η = 0.01,  A is red,  B is green, and  C is blue. Figure S6: Fractionation of monodisperse ternary (component *A*, *B*, and *C*) non-additive hard spheremixture with size ratio *q_AB_* = *σ_A_/σ_B_* = 1*/*4 and *q_AC_* = *σ_A_/σ_C_* = 1*/*3, with non-additivity parameters: ∆*_AB_* = 0.1, ∆*_AC_* = 0.1, and ∆*_BC_* = 0, fixed parent phase: *η*(0.05, 0.20, 0.10), adjusting A, *B*, resp. *C* with *η* = 0.01,  *A* is red,  *B* is green, and  *C* is blue. Figure S7: Fractionation of monodisperse ternary (component *A*, *B*, and *C*) non-additive hard sphere mixture with size ratio *q_AB_* = *σ_A_/σ_B_* = 1*/*4 and *q_AC_* = *σ_A_/σ_C_* = 1*/*3, with non-additivity parameters: ∆*_AB_* = 0.1, ∆*_AC_* = −0.1, and ∆*_BC_* = 0.1, fixed parent phase: *η*(0.05, 0.20, 0.10), adjusting *A*, *B*, resp. *C* with *η* = 0.01,  *A* is red,  *B* is green, and  *C* is blue. Figure S8: Fractionation of monodisperse ternary (component *A*, *B*, and *C*) non-additive hard sphere mixture with size ratio *q_AB_* = *σ_A_/σ_B_* = 1/4 and *q_AC_ = σ_A_/σ_C_* = 1/3, with non-additivity parameters: ∆*_AB_* = 0.1, ∆*_AC_* = 0, and ∆*_BC_* = 0.1, fixed parent phase: *η*(0.05, 0.20, 0.10), adjusting *A*, *B*, resp. *C* with *η* = 0.01,  *A* is red,  *B* is green, and  *C* is blue. Figure S9: Fractionation of monodisperse ternary (component *A*, *B*, and *C*) non-additive hard sphere mixture with size ratio *q_AB_* = *σ_A_/σ_B_* = 1*/*4 and *q_AC_* = *σ_A_/σ_C_* = 1*/*3, with non-additivity parameters: ∆*_AB_* = 0.1, ∆*_AC_* = 0.1, and ∆*_BC_* = 0.1, fixed parent phase: *η*(0.05, 0.20, 0.10), adjusting *A*, *B*, resp. *C* with *η* = 0.01,  *A* is red,  *B* is green, and  *C* is blue. Table S1: Fractionation of monodisperse ternary (component *A*, *B*, and *C*) non-additive hard sphere mixtures with size ratio *q_AB_* = *σ_A_/σ_B_* = 1*/*4 and *q_AC_* = *σ_A_/σ_C_* = 1*/*3, with non-additivity parameters: ∆*_AB_* = 0.1, ∆*_AC_* and ∆*_BC_* varying from −0.1 to 0.1, label referring to Figure 3, Figure 4, Figure 5, Figure 6, Figure 7, Figure 8, Figure 9, Figure 10 and Figure 11, fixed parent phase: *η*(0.05, 0.20, 0.10), see also Figure 12a. Table S2: Fractionation of monodisperse ternary (component *A*, *B*, and *C*) non-additive hard sphere mixtures with size ratio *q_AB_* = *σ_A_/σ_B_* = 1*/*4 and *q_AC_* = *σ_A_/σ_C_* = 1*/*3, with non-additivity parameters: ∆*_AB_* = 0.1, ∆*_AC_* and ∆*_BC_* variying from −0.1 to 0.1, label refering to Figure 3, Figure 4, Figure 5, Figure 6, Figure 7, Figure 8, Figure 9, Figure 10 and Figure 11, fixed parent phase: *η*(0.05, 0.20, 0.25), see also Figure 12b. Table S3: Fractionation of monodisperse ternary (component *A*, *B*, and *C*) non-additive hard sphere mixtures with size ratio *q_AB_* = *σ_A_/σ_B_* = 1*/*4 and *q_AC_* = *σ_A_/σ_C_* = 1*/*3, with non-additivity parameters: ∆*_AB_* = 0.1, ∆*_AC_* and ∆*_BC_* variying from −0.1 to 0.1, label refering to Figure 3, Figure 4, Figure 5, Figure 6, Figure 7, Figure 8, Figure 9, Figure 10 and Figure 11, fixed parent phase: *η*(0.10, 0.20, 0.10), see also Figure 13a. Table S4: Fractionation of monodisperse ternary (component *A*, *B*, and *C*) non-additive hard sphere mixtures with size ratio *q_AB_* = *σ_A_/σ_B_* = 1*/*4 and *q_AC_* = *σ_A_/σ_C_* = 1*/*3, with non-additivity parameters: ∆*_AB_* = 0.1, ∆*_AC_* and ∆*_BC_* variying from −0.1 to 0.1, label refering to Figure 3, Figure 4, Figure 5, Figure 6, Figure 7, Figure 8, Figure 9, Figure 10 and Figure 11, fixed parent phase: *η*(0.05, 0.10, 0.10), see also Figure 13b.
