# Supplementary material for: Ternary Mixtures of Hard Spheres and Their Multiple Separated Phases
Source: Molecules. 2023 Nov 28;28(23):7817. doi: 10.3390/molecules28237817 (PMC10707887; doi:10.3390/molecules28237817)
Supplement: Supplementary file 1 [file molecules-28-07817-s001.zip › molecules-2592320-supplementary.pdf]

Supplementary

Luka Sturtewagen and Erik van der Linden

Laboratory of Physics and Physical Chemistry of Foods,

Wageningen University, Bornse Weilanden 9, 6708 WG Wageningen, The Netherlands

TABLES

Table S1. Fractionation of monodisperse ternary (component A, B, and C) non-additive hard sphere mixtures with size ratio $q_{AB} = \sigma_A/\sigma_B = 1/4$ and $q_{AC} = \sigma_A/\sigma_C = 1/3$, with non-additivity parameters: $\Delta_{AB} = 0.1$, Δ_{AC} and Δ_{BC} varying from −0.1 to 0.1, label referring to **Figure 3-11**, fixed parent phase: $\eta(0.05, 0.20, 0.10)$, see also Figure 12a.

Mixture	Top phase	Bottom phase
4.3	η (0.061, 0.054, 0.104)<br>α: 0.733	η (0.021, 0.602, 0.089)<br>α: 0.267
4.4	η (0.060, 0.046, 0.117)<br>α: 0.770	η (0.017, 0.716, 0.043)<br>α: 0.223
4.5	η (0.063, 0.044, 0.087)<br>α: 0.682	η (0.022, 0.534, 0.127)<br>α: 0.318
4.6	η (0.060, 0.055, 0.107)<br>α: 0.749	η (0.020, 0.633, 0.080)<br>α: 0.251
4.7	η (0.069, 0.020, 0.049)<br>α: 0.611	η (0.020, 0.483, 0.180)<br>α: 0.389
4.8	η (0.066, 0.034, 0.073)<br>α: 0.622	η (0.024, 0.473, 0.145)<br>α: 0.378
4.9	η (0.060, 0.027, 0.126)<br>α: 0.779	η (0.014, 0.808, 0.010)<br>α: 0.222
4.10	η (0.059, 0.040, 0.121)<br>α: 0.790	η (0.015, 0.803, 0.020)<br>α: 0.210
4.11	η (0.059, 0.057, 0.113)<br>α: 0.787	η (0.017, 0.731, 0.052)<br>α: 0.213

Table S2. Fractionation of monodisperse ternary (component A, B, and C) non-additive hard sphere mixtures with size ratio $q_{AB} = \sigma_A/\sigma_B = 1/4$ and $q_{AC} = \sigma_A/\sigma_C = 1/3$, with non-additivity parameters: $\Delta_{AB} = 0.1$, Δ_{AC} and Δ_{BC} variying from −0.1 to 0.1, label refering to **Figure 3-11**, fixed parent phase: η(0.05, 0.20, 0.25), see also **Figure 12b**

**Mixture**	**Top phase**	**Bottom phase**
4.3	η (0.060, 0.054, 0.257)<br>α: 0.742	η (0.020, 0.619, 0.230)<br>α: 0.258
4.4	η (0.059, 0.040, 0.284)<br>α: 0.813	η (0.013, 0.894, 0.105)<br>α: 0.187
4.5	η (0.065, 0.037, 0.223)<br>α: 0.644	η (0.023, 0.495, 0.298)<br>α: 0.356
4.6	η (0.059, 0.057, 0.265)<br>α: 0.788	η (0.018, 0.715, 0.195)<br>α: 0.217
4.7	η (0.084, 0.003, 0.086)<br>α: 0.468	η (0.020, 0.373, 0.394)<br>α: 0.532
4.8	η (0.085, 0.007, 0.103)<br>α: 0.406	η (0.026, 0.332, 0.350)<br>α: 0.594
4.9	η (0.059, 0.012, 0.300)<br>α: 0.827	η (0.009, 1.097, 0.013)<br>α: 0.173
4.10	η (0.058, 0.023, 0.294)<br>α: 0.838	η (0.009, 1.116, 0.023)<br>α: 0.162
4.11	η (0.057, 0.048, 0.284)<br>α: 0.855	η (0.011, 1.095, 0.050)<br>α: 0.145

Table S3. Fractionation of monodisperse ternary (component A, B, and C) non-additive hard sphere mixtures with size ratio $q_{AB} = \sigma_A/\sigma_B = 1/4$ and $q_{AC} = \sigma_A/\sigma_C = 1/3$, with non-additivity parameters: $\Delta_{AB} = 0.1$, Δ_{AC} and Δ_{BC} variiying from −0.1 to 0.1, label refering to **Figure 3-11**, fixed parent phase: η(0.10, 0.20, 0.10), see also Figure 13a

Mixture	Top phase	Middle phase	Bottom phase
4.3	η (0.116, 0.001, 0.102)<br>α: 0.852		η (0.009, 1.345, 0.089)<br>α: 0.148
4.4	η (0.115, 0.001, 0.113)<br>α: 0.864		η (0.007, 1.461, 0.015)<br>α: 0.136
4.5	η (0.119, 0.001, 0.070)<br>α: 0.823		η (0.010, 1.125, 0.240)<br>α: 0.177
4.6	η (0.116, 0.001, 0.104)<br>α: 0.854		η (0.008, 1.367, 0.080)<br>α: 0.146
4.7	η (0.126, 0.000, 0.013)<br>α: 0.774		η (0.010, 0.886, 0.398)<br>α: 0.226
4.8	η (0.127, 0.000, 0.026)<br>α: 0.766		η (0.013, 0.855, 0.344)<br>α: 0.234
4.9	η (0.114, 0.001, 0.115)<br>α: 0.866		η (0.007, 1.489, 0.001)<br>α: 0.134
4.10	η (0.114, 0.001, 0.114)<br>α: 0.869		η (0.007, 1.517, 0.005)<br>α: 0.131
4.11	η (0.123, 0.001, 0.051)<br>α: 0.778	η (0.035, 0.044, 0.636)<br>α: 0.087	η (0.008, 1.451, 0.036)<br>α: 0.135

Table S4. Fractionation of monodisperse ternary (component *A*, *B*, and *C*) non-additive hard sphere mixtures with size ratio $q_{AB} = \sigma_A/\sigma_B = 1/4$ and $q_{AC} = \sigma_A/\sigma_C = 1/3$, with non-additivity parameters: $\Delta_{AB} = 0.1$, Δ_{AC} and Δ_{BC} variying from −0.1 to 0.1, label refering to **Figure 3-11**, fixed parent phase: η(0.05, 0.10, 0.10), see also **Figure 13b**

**Mixture**	**Top phase**	**Bottom phase**
4.3		η (0.050, 0.100, 0.100)<br>α: 1
4.4	η (0.051, 0.083, 0.102)<br>α: 0.964	η (0.021, 0.561 0.050)<br>α: 0.036
4.5		η (0.050, 0.100, 0.100)<br>α: 1
4.6		η (0.050, 0.100, 0.100)<br>α: 1
4.7	η (0.056, 0.047, 0.082)<br>α: 0.803	η (0.027, 0.315, 0.172)<br>α: 0.197
4.8	η (0.051, 0.091, 0.099)<br>α: 0.956	η (0.032, 0.289, 0.132)<br>α: 0.044
4.9	η (0.053, 0.047, 0.108)<br>α: 0.915	η (0.016, 0.675, 0.014)<br>α: 0.085
4.10	η (0.052, 0.067, 0.104)<br>α: 0.944	η (0.018, 0.661, 0.026)<br>α: 0.056
4.11	η (0.050, 0.098, 0.100)<br>α: 0.996	η (0.021, 0.573, 0.057)<br>α: 0.004

FRACTIONATION

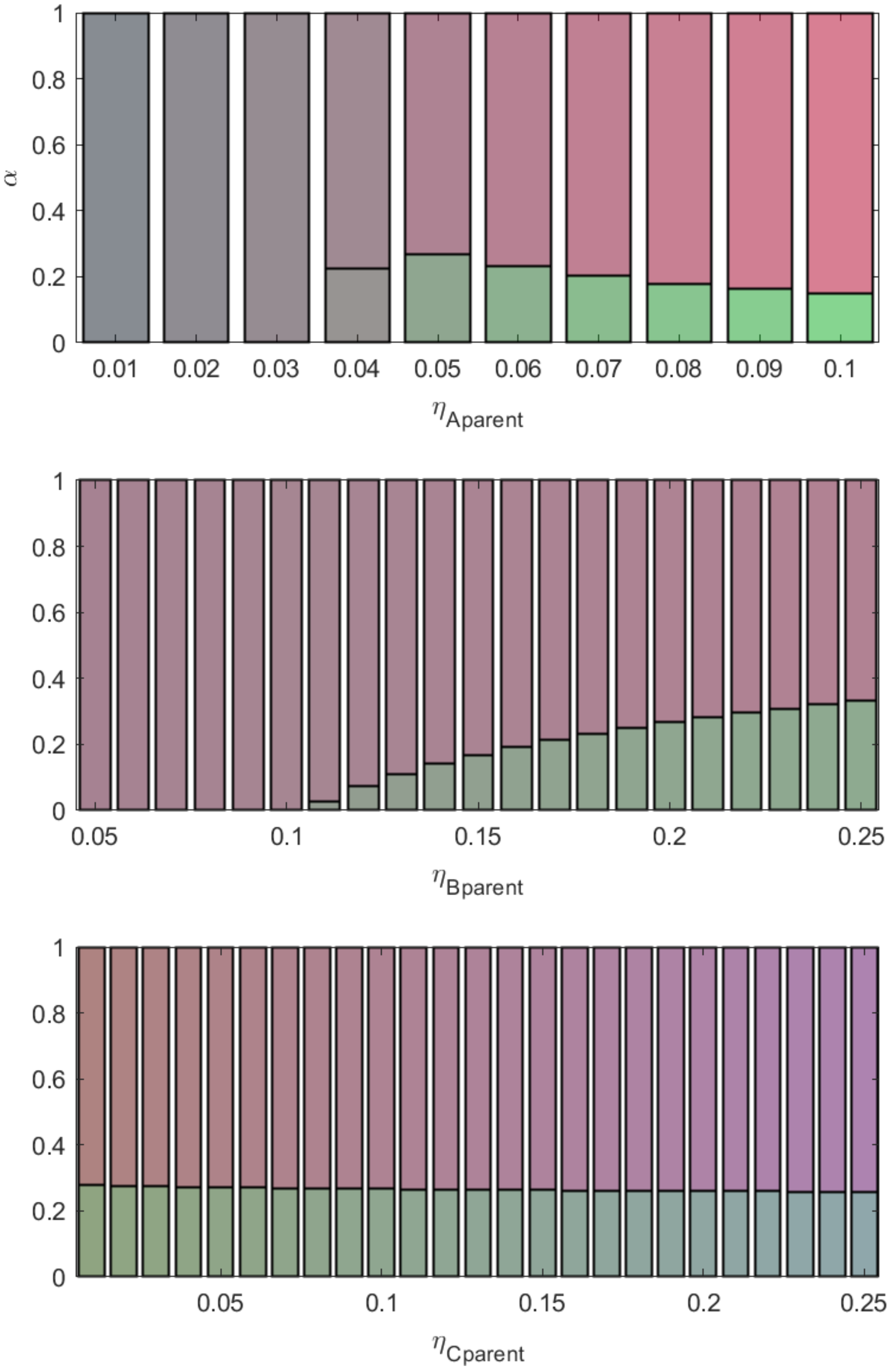

Figure S1. Fractionation of monodisperse ternary (component A, B, and C) non-additive hard sphere mixture with size ratio $q_{AB} = \sigma_A/\sigma_B = 1/4$ and $q_{AC} = \sigma_A/\sigma_C = 1/3$, with non-additivity parameters: $\Delta_{AB} = 0.1$, $\Delta_{AC} = -0.1$, and $\Delta_{BC} = -0.1$, fixed parent phase: $\eta(0.05, 0.20, 0.10)$, adjusting A, B, resp. C with $\eta = 0.01$, A is red, B is green , C is blue

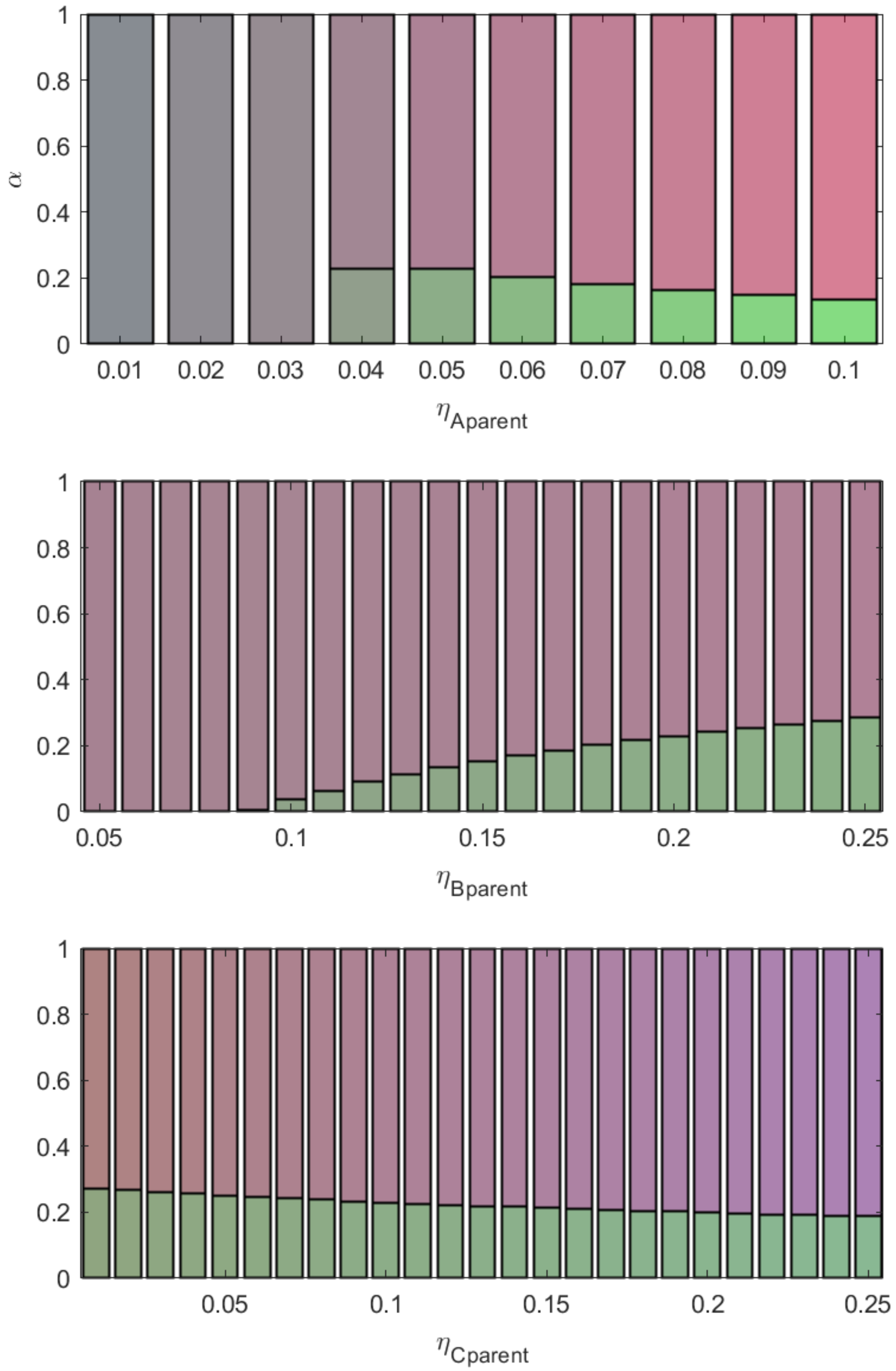

Figure S2. Fractionation of monodisperse ternary (component A, B, and C) non-additive hard sphere mixture with size ratio $q_{AB} = \sigma_A/\sigma_B = 1/4$ and $q_{AC} = \sigma_A/\sigma_C = 1/3$, with non-additivity parameters: $\Delta_{AB} = 0.1$, $\Delta_{AC} = -0.1$, and $\Delta_{BC} = 0$, fixed parent phase: $\eta(0.05, 0.20, 0.10)$, A is red, B is green , C is blue

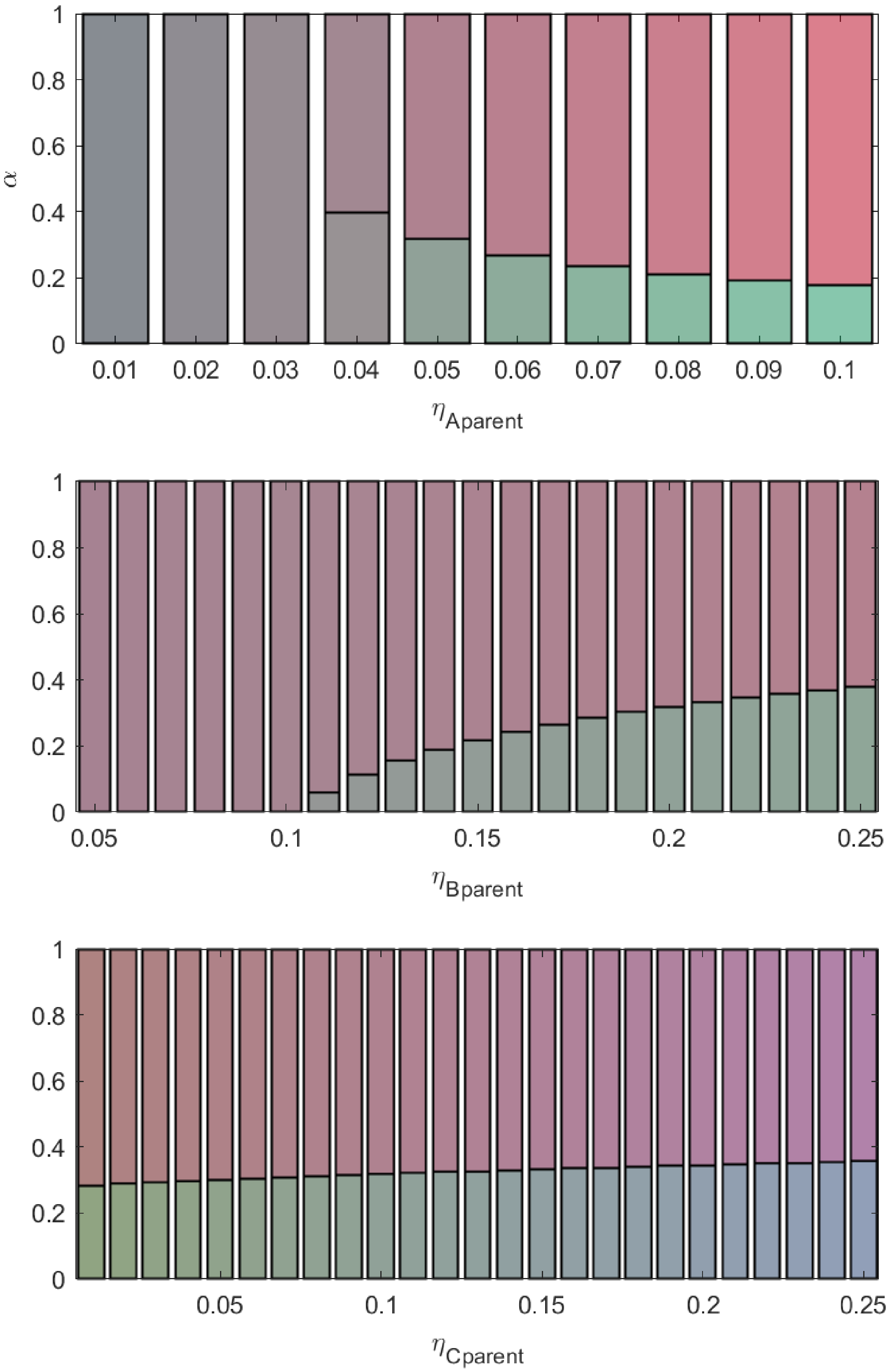

Figure S3. Fractionation of monodisperse ternary (component A, B, and C) non-additive hard sphere mixture with size ratio $q_{AB} = \sigma_A/\sigma_B = 1/4$ and $q_{AC} = \sigma_A/\sigma_C = 1/3$, with non-additivity parameters: $\Delta_{AB} = 0.1$, $\Delta_{AC} = 0$, and $\Delta_{BC} = -0.1$, fixed parent phase: $\eta(0.05, 0.20, 0.10)$, A is red, B is green, C is blue

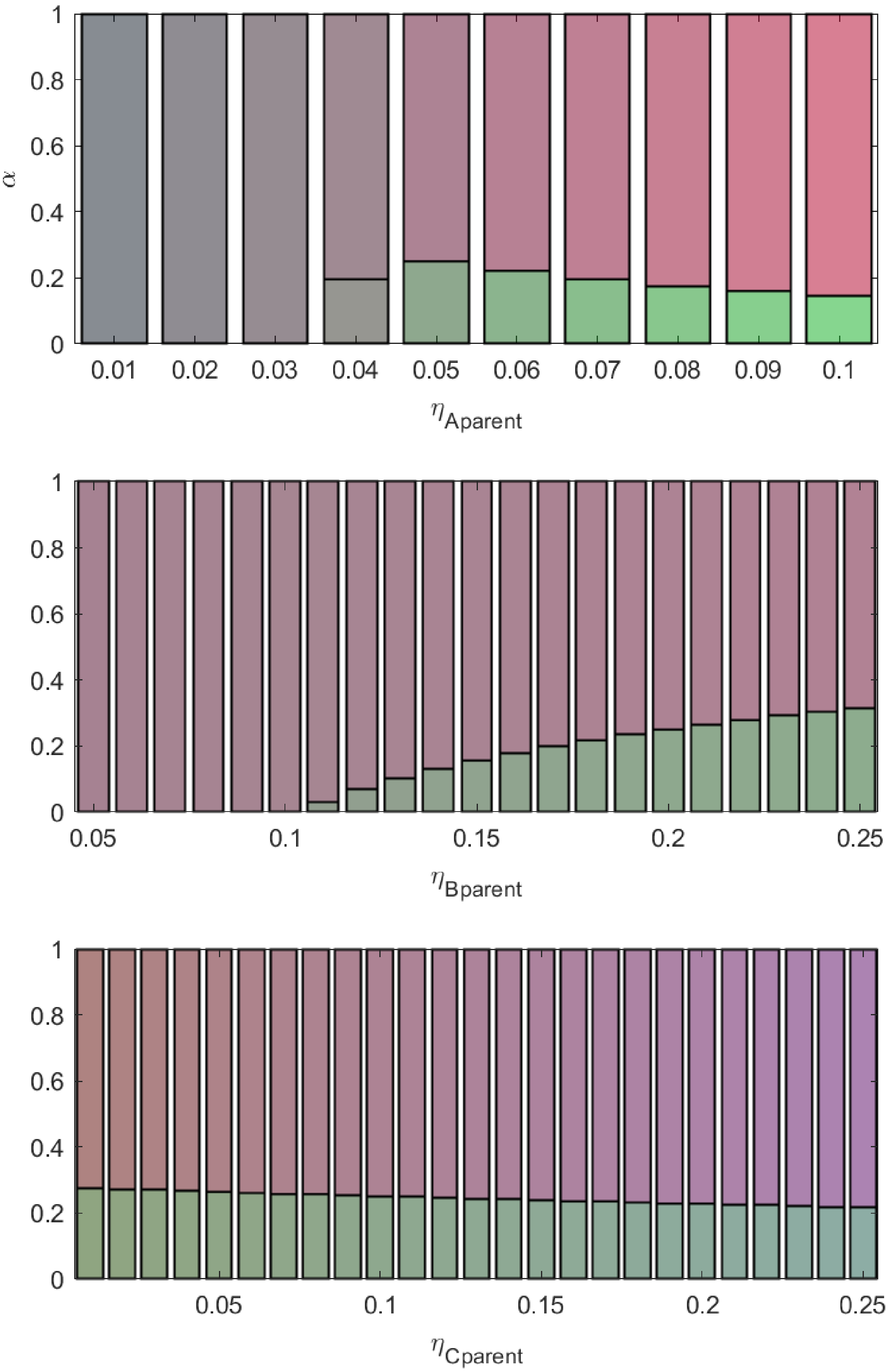

Figure S4. Fractionation of monodisperse ternary (component A, B, and C) non-additive hard sphere mixture with size ratio $q_{AB} = \sigma_A/\sigma_B = 1/4$ and $q_{AC} = \sigma_A/\sigma_C = 1/3$, with non-additivity parameters: $\Delta_{AB} = 0.1$, $\Delta_{AC} = 0$, and $\Delta_{BC} = 0$, fixed parent phase: $\eta(0.05, 0.20, 0.10)$, adjusting A, B, resp. C with $\eta = 0.01$, A is red, B is green, and C is blue

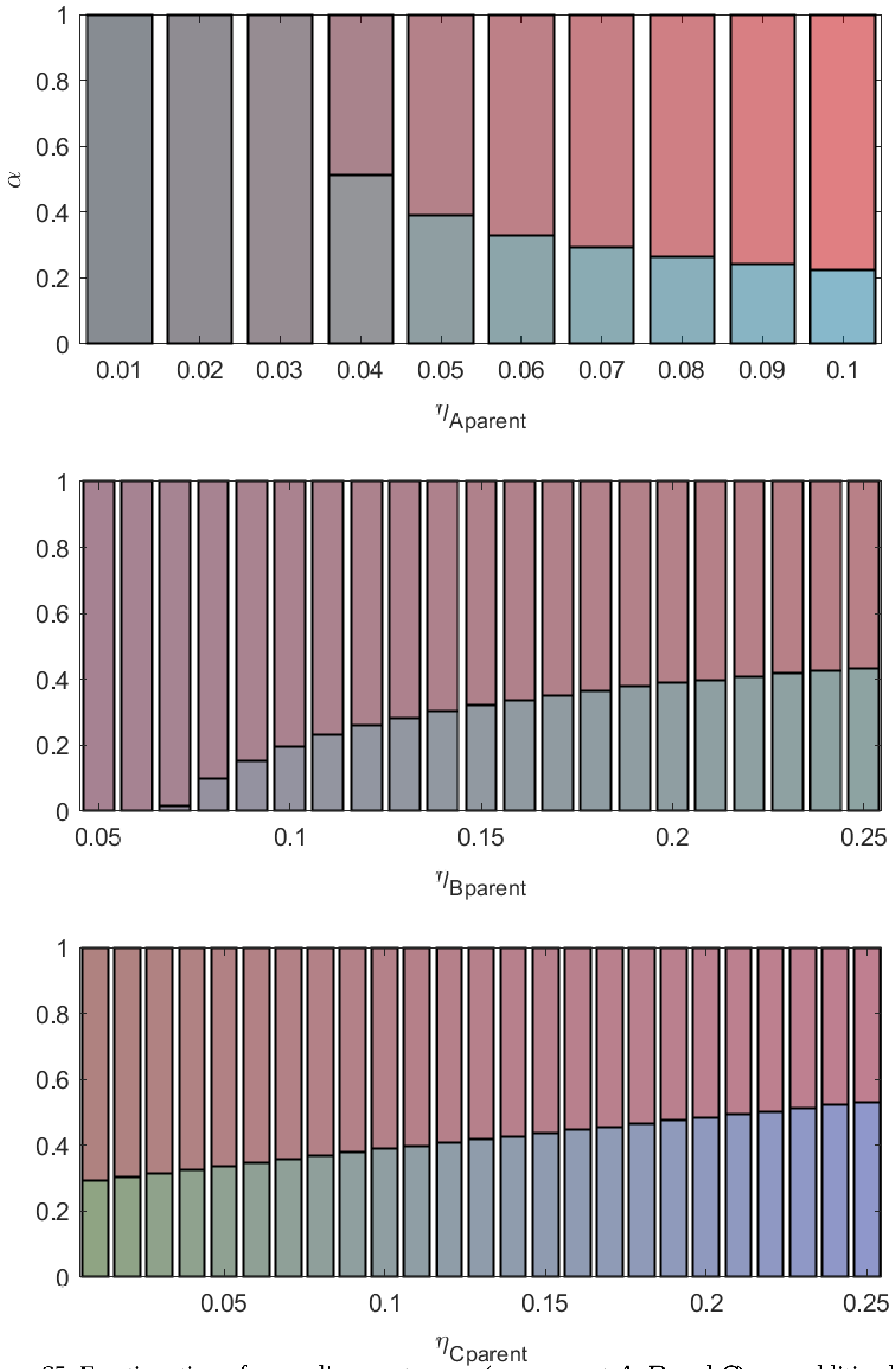

Figure S5. Fractionation of monodisperse ternary (component A, B, and C) non-additive hard sphere mixture with size ratio $q_{AB} = \sigma_A/\sigma_B = 1/4$ and $q_{AC} = \sigma_A/\sigma_C = 1/3$, with non-additivity parameters: $\Delta_{AB} = 0.1$, $\Delta_{AC} = 0.1$, and $\Delta_{BC} = -0.1$, fixed parent phase: $\eta(0.05, 0.20, 0.10)$, adjusting A, B, resp. C with $\eta = 0.01$, A is red, B is green, and C is blue

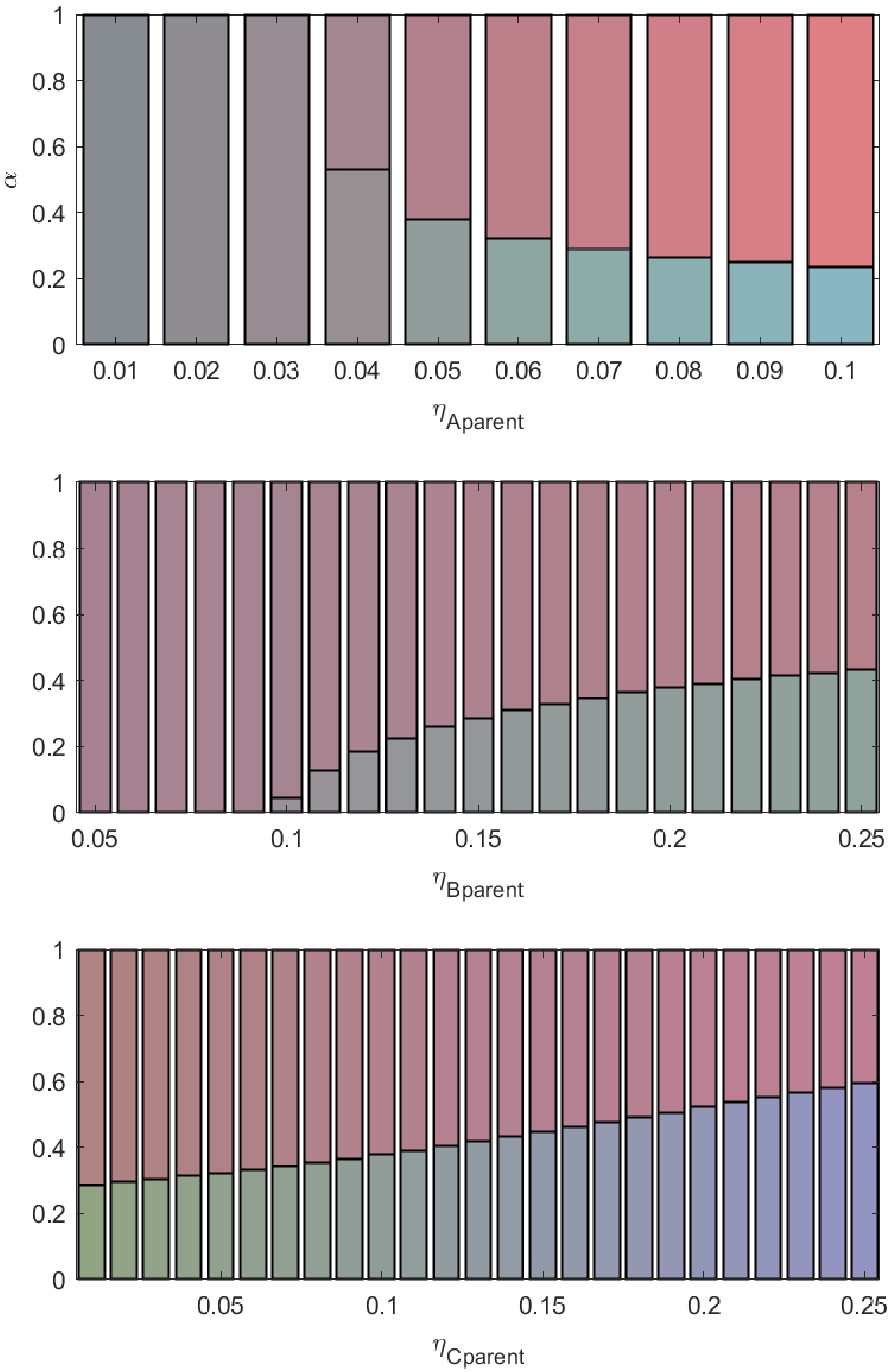

Figure S6. Fractionation of monodisperse ternary (component A, B, and C) non-additive hard spheremixture with size ratio $q_{AB} = \sigma_A/\sigma_B = 1/4$ and $q_{AC} = \sigma_A/\sigma_C = 1/3$, with non-additivity parameters: $\Delta_{AB} = 0.1$, $\Delta_{AC} = 0.1$, and $\Delta_{BC} = 0$, fixed parent phase: $\eta(0.05, 0.20, 0.10)$, adjusting A, B, resp. C with $\eta = 0.01$, A is red, B is green, and C is blue

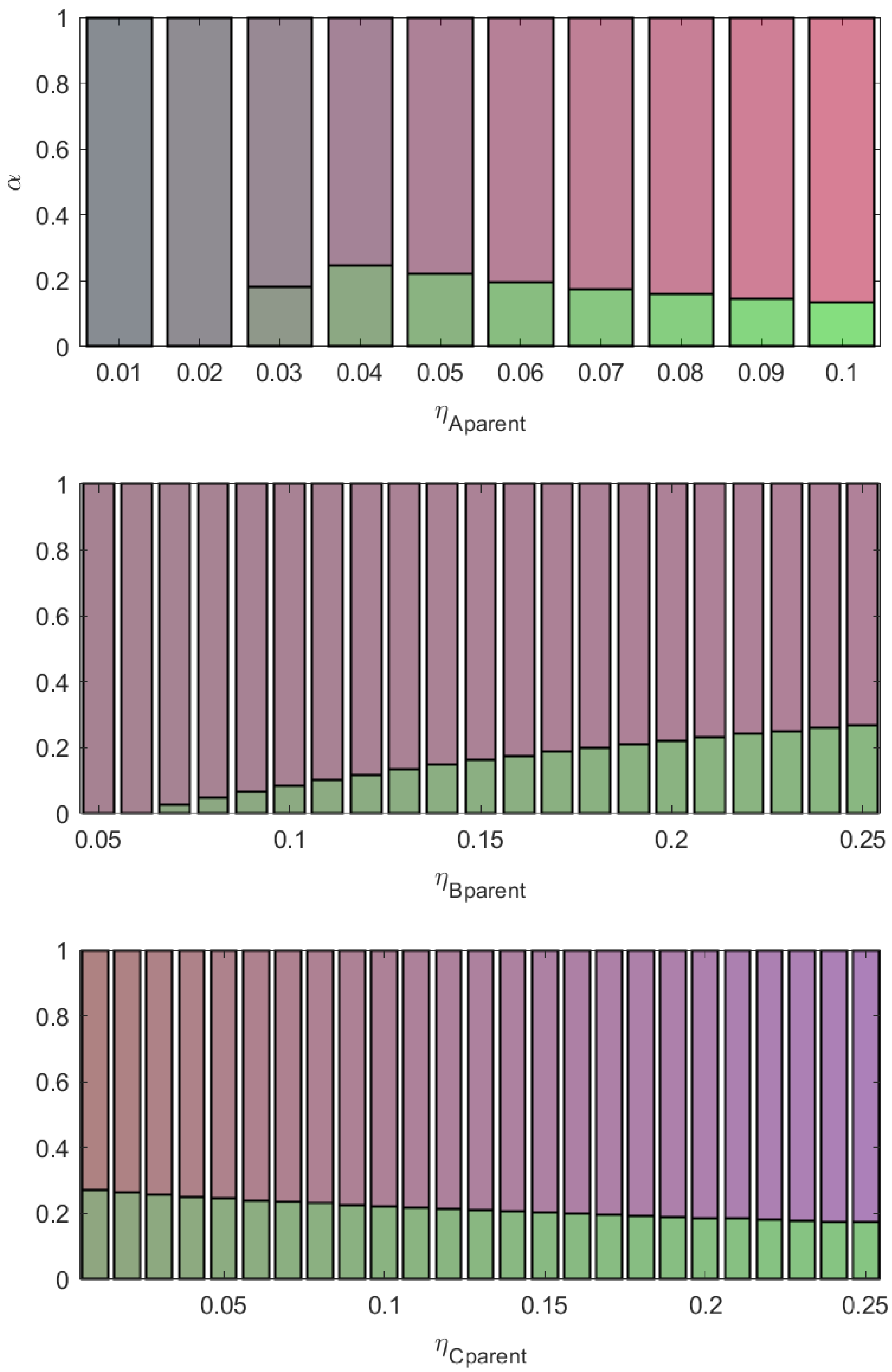

Figure S7. Fractionation of monodisperse ternary (component A, B, and C) non-additive hard sphere mixture with size ratio $q_{AB} = \sigma_A/\sigma_B = 1/4$ and $q_{AC} = \sigma_A/\sigma_C = 1/3$, with non-additivity parameters: $\Delta_{AB} = 0.1$, $\Delta_{AC} = -0.1$, and $\Delta_{BC} = 0.1$, fixed parent phase: $\eta(0.05, 0.20, 0.10)$, adjusting A, B, resp. C with $\eta = 0.01$, A is red, B is green, and C is blue

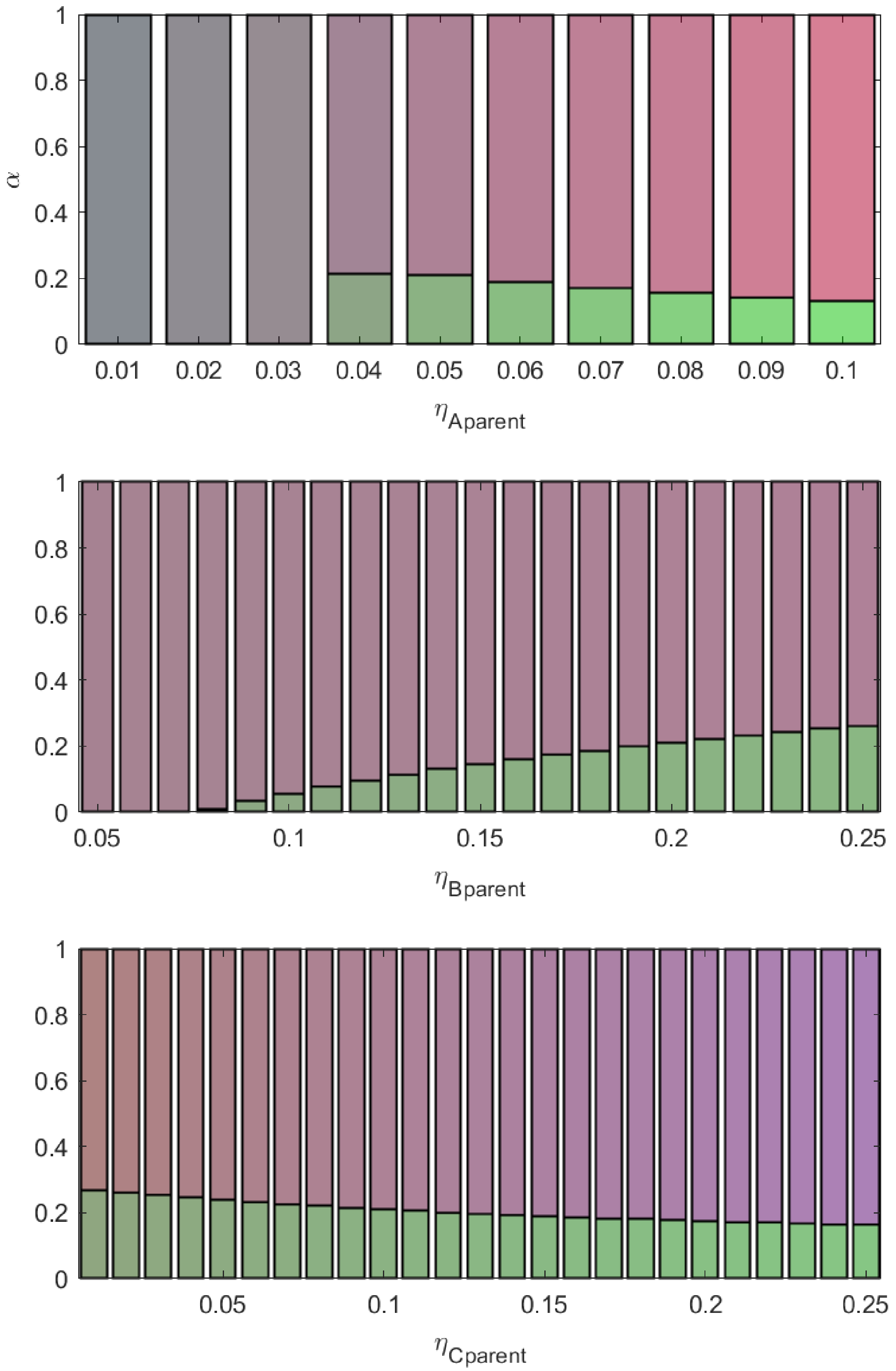

Figure S8. Fractionation of monodisperse ternary (component A, B, and C) non-additive hard sphere mixture with size ratio $q_{AB} = \sigma_A/\sigma_B = 1/4$ and $q_{AC} = \sigma_A/\sigma_C = 1/3$, with non-additivity parameters: $\Delta_{AB} = 0.1$, $\Delta_{AC} = 0$, and $\Delta_{BC} = 0.1$, fixed parent phase : $\eta(0.05, 0.20, 0.10)$, adjusting A, B, resp. C with $\eta = 0.01$, A is red , B is green, and C is blue

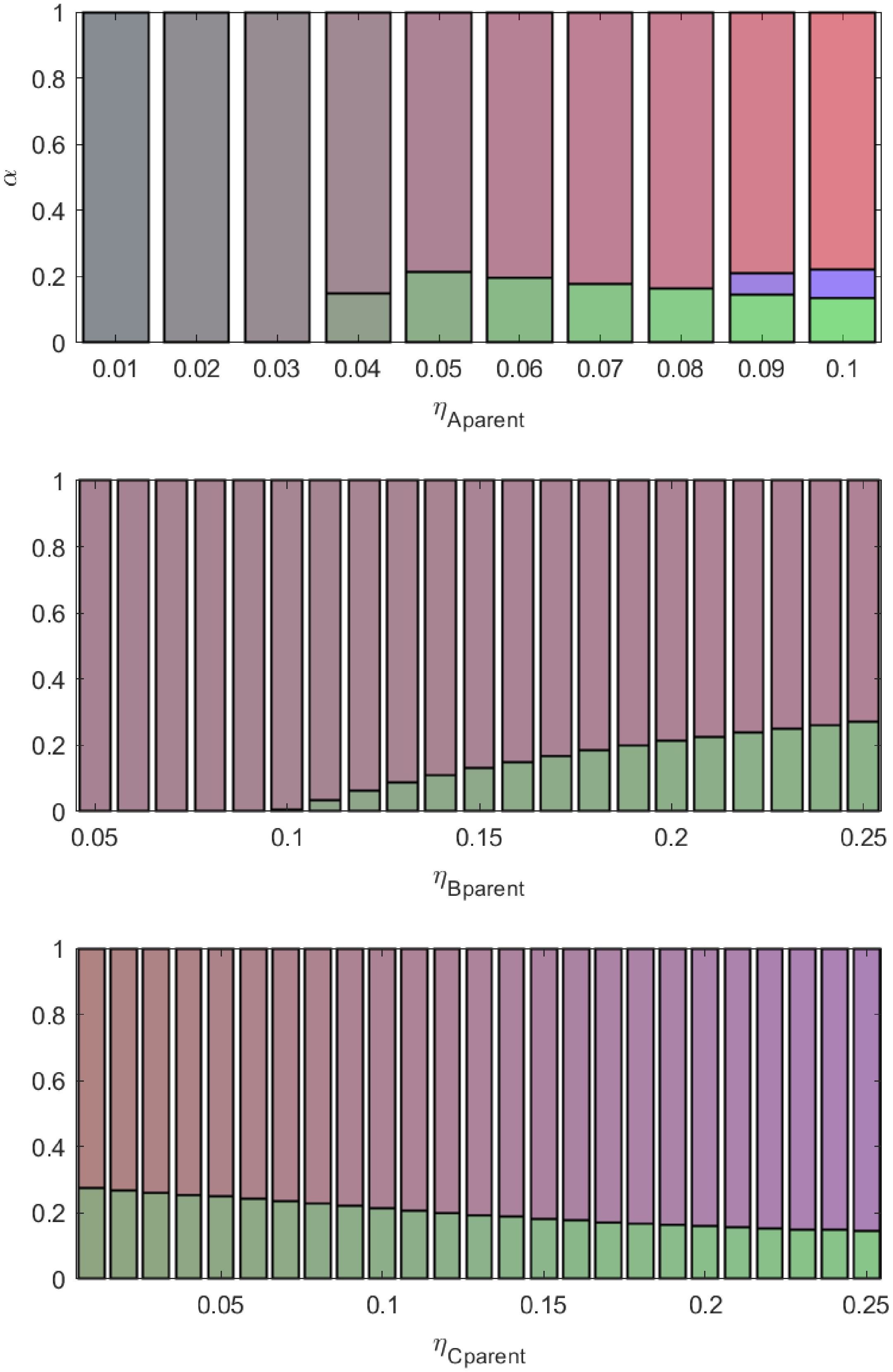

Figure S9. Fractionation of monodisperse ternary (component *A*, *B*, and *C*) non-additive hard sphere mixture with size ratio $q_{AB} = \sigma_A/\sigma_B = 1/4$ and $q_{AC} = \sigma_A/\sigma_C = 1/3$, with non-additivity parameters: $\Delta_{AB} = 0.1$, $\Delta_{AC} = 0.1$, and $\Delta_{BC} = 0.1$, fixed parent phase: $\eta(0.05, 0.20, 0.10)$, adjusting *A*, *B*, resp. *C* with $\eta = 0.01$, *A* is red, *B* is green, and *C* is blue
